# Serpina3n deficiency exacerbates sepsis‐induced myocardial dysfunction by disrupting fibroblast–cardiomyocyte crosstalk

**DOI:** 10.1002/ctm2.70898

**Published:** 2026-11-14

**Authors:** Xunuo Wang, Yufeng Yan, Aiqing Kong, Xiya Li, Hao Xie, Zuoying Hu, Guangfeng Zuo

**Affiliations:** ^1^ Department of Cardiology, Nanjing First Hospital Nanjing Medical University Nanjing China

**Keywords:** fibroblast‒cardiomyocyte crosstalk, JAK–STAT, sepsis‐induced myocardial dysfunction, serine protease inhibitor

## Abstract

**Background:**

Sepsis‐induced myocardial dysfunction (SIMD) is a key contributor to death among individuals with sepsis. Serpina3n (a serine protease inhibitor) contributes critically to various cardiovascular diseases. However, whether Serpina3n is involved in SIMD and its underlying mechanisms remain unclear.

**Methods:**

A SIMD model was established by either an intraperitoneal injection of lipopolysaccharide (LPS) or caecal ligation and puncture (CLP) in mice. Single‐cell RNA sequencing (scRNA‐seq) was carried out to examine Serpina3n expression and its cellular localisation in mouse hearts. Serpina3n expression was also examined in LPS‐treated neonatal mouse cardiac fibroblasts (NMCFs) and neonatal mouse cardiomyocytes (NMCMs). Global Serpina3n knockout (KO) (Serpina3n^−/−^) and fibroblast‐specific Serpina3n‐deficient (Serpina3n^flox/flox^, Col1a2‐iCre) mouse models were used for functional validation in vivo.

**Results:**

Serpina3n expression was notably up‐regulated in the hearts of SIMD mice, and it was enriched mainly in cardiac fibroblasts (CFs). Consistent with the scRNA‐seq data, Serpina3n expression was markedly increased in LPS‐treated NMCFs but not in NMCMs. Furthermore, both global Serpina3n KO and fibroblast‐specific Serpina3n deletion exacerbated cardiac inflammation and dysfunction, facilitated the progression of SIMD and ultimately increased mortality. Mechanistically, LPS stimulation promoted the secretion of Serpina3n from CFs, which acted on cardiomyocytes (CMs) in a paracrine manner, thereby attenuating the expression of proteins related to the Janus kinase/signal transducer and activator of transcription signalling pathway and reducing cardiac inflammatory cell infiltration to ultimately exert a cardioprotective effect. These processes were disrupted by fibroblast‐specific Serpina3n deficiency.

**Conclusions:**

Collectively, these findings indicate that CF‐derived Serpina3n mediates CF–CM crosstalk and plays an essential protective role in SIMD, providing mechanistic insights and a potential candidate target for further preclinical investigations of SIMD.

## Key points

- LPS induces STAT3 phosphorylation and Serpina3n up‐regulation in cardiac fibroblasts.
- Fibroblast‐derived Serpina3n acts on cardiomyocytes via paracrine signalling.
- Serpina3n inhibits the JAK–STAT pathway in cardiomyocytes.
- Suppression of inflammatory cytokine production confers cardioprotection in SIMD.

## Graphical abstract

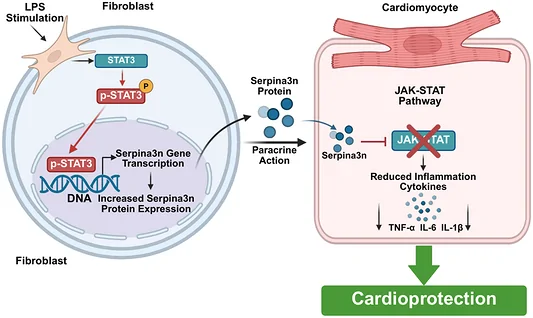

## 1 INTRODUCTION

Sepsis is derived from an infection that induces a systemic inflammatory cascade. It consistently accounts for a high proportion of fatalities within intensive care units worldwide.^1^, ^2^ Systemic inflammatory response syndrome, septic shock, multiple organ dysfunction syndrome (MODS) and other associated disorders are potential complications of this condition.^3^, ^4^ Among the various organ injuries induced by sepsis, cardiovascular dysfunction is associated with the incidence of MODS.^5^ Sepsis‐induced cardiovascular dysfunction, termed sepsis‐induced myocardial dysfunction (SIMD), has emerged as a critical determinant of a poor sepsis prognosis and is linked to increased mortality of patients with sepsis.^6^, ^7^, ^8^ Thus, the exploration of potential therapeutic targets for SIMD is urgent.

The pathophysiological mechanism of SIMD is complex and multifactorial. Its central pathogenesis involves dysregulated host immune responses that trigger a cytokine storm dominated by the triad of interleukin‐1β (IL‐1β), tumour necrosis factor‐α (TNF‐α) and IL‐6.^9^, ^10^ Pro‐inflammatory cytokines act as pivotal modulating factors that govern the initiation and progression of SIMD.^11^ Upon stimulation by the abovementioned pro‐inflammatory cytokines, acute phase proteins (APPs), such as serum amyloid A and C‐reactive protein, are produced and secreted in large amounts by hepatocytes within hours.^12^, ^13^, ^14^ APPs serve as conventional biological indicators for sepsis, given that their concentrations notably increase in the blood serum of individuals diagnosed with this condition.^12^, ^13^, ^14^, ^15^ Although APPs are associated with inflammation and sepsis and are thought to potentially predict the prognosis of SIMD, it remains unclear whether these APPs contribute to the initiation and progression of SIMD. Given the higher APP levels in the serum of patients with SIMD,^16^ theoretically, APPs may act as a connecting mediator of the causal relationship between inflammatory responses and SIMD. Thus, evidence that supports this conclusion needs to be provided immediately.

SerpinA3, a serine protease inhibitor, is an APP.^17^ As an important member of the serine protease inhibitor family, SerpinA3/Serpina3n (murine ortholog of human SerpinA3) can attenuate tissue injury, restrict excessive immune cell infiltration and modulate inflammatory signalling pathways by inhibiting serine proteases such as granzyme B, leukocyte elastase and cathepsin G.^17^, ^18^, ^19^ These effects contribute to tissue protection in models of wound healing, aortic aneurysm and neuropathic pain.^20^, ^21^, ^22^ Notably, the functions of SerpinA3/Serpina3n are context‐dependent, being protective in tissue repair and ischemic injury, but pro‐inflammatory or pro‐fibrotic in neuroinflammation and allergic airway inflammation.^23^, ^24^ SerpinA3 plays a crucial role in cardiovascular disorders; for instance, it contributes to prognostic stratification in new‐onset or worsening heart failure,^25^ and Serpina3n participates in heart repair after acute myocardial infarction by modulating inflammatory responses and fibrosis progression.^26^, ^27^ Both publicly available GEO records (GSE267388, GSE215955 and GSE229925) and our RNA sequencing demonstrated a significant rise in Serpina3n mRNA expression within the hearts of SIMD mice. Furthermore, our results indicated that both global Serpina3n KO (Serpina3n^−/−^) and fibroblast‐specific Serpina3n deletion (Serpina3n^flox/flox^, Col1a2‐iCre) exacerbated cardiac inflammatory cell infiltration and dysfunction, facilitated SIMD progression and ultimately increased mortality. Based on the literature and the abovementioned findings, Serpina3n exerts a cardioprotective effect on SIMD. Yet, the mechanistic role of Serpina3n in SIMD is still poorly understood. Therefore, we aimed to investigate its underlying mechanisms and sought to provide novel therapeutic targets for enhancing the outcomes of patients suffering from SIMD.

## 2 MATERIALS AND METHODS

### 2.1 Experimental animals

All experimental manipulations conformed to applicable ethical norms. Animals were housed within a conventional animal facility under a 12‐h light–dark cycle, with unrestricted access to drinking water and rodent chow. Additionally, the housing environment was kept at 22 ± 2°C with relative humidity stabilised within the range of 50 ± 10%. Serpina3n knockout (KO) (Cas9) C57BL/6 mice and Serpina3n‐Col1a2‐iCre (Cas9) C57BL/6 mice were obtained from Cyagen Biosciences, Inc. (Suzhou, Jiangsu, China), and GemPharmatech LLC (Nanjing, Jiangsu, China) supplied male wild‐type (WT) C57BL/6 mice with body masses of 20–25 g at 6–8 weeks of age. Neonatal C57BL/6 mice (males and females; aged 0–2 days) were obtained from GemPharmatech for use in cell culture studies in vitro. Each figure legend specifies the number of mice for the respective experiment.

### 2.2 Endotoxemia model

Lipopolysaccharide (LPS; Sigma‒Aldrich, St. Louis, Missouri, USA) was dissolved in sterile phosphate‐buffered saline (PBS) and given intraperitoneally to induce endotoxemia. Mice assigned to the control group were intraperitoneally administered an equal volume of PBS, which served as the vehicle for LPS (10 mg/kg). Animals were sacrificed with excess sodium pentobarbital (150 mg/kg) 12 h after LPS was administered, and the hearts were perfused with PBS to remove blood contaminants. Afterwards, heart tissue samples were collected and stored. Mice in the survival study were monitored for 72 h following intraperitoneal LPS injection.

### 2.3 Caecal ligation and puncture model

The caecal ligation and puncture (CLP) model was constructed using the method reported earlier.^28^, ^29^ After isoflurane (5% for induction, 1.5–2% for maintenance) was administered in 100% oxygen via a nasal cone, the abdomens were fully exposed. A midline laparotomy was performed to visualise the cecum, which was then occluded with 4‑0 silk sutures at a point 50% from the tip towards the base. Next, the ligated cecum was perforated once using a 22‑G needle via a through‑and‑through approach, after which slight faecal extrusion was performed to check for patency. In the sham‐operated animals, the abdominal incision and intestinal manipulation were performed without ligation or puncture. All the animals received subcutaneous injections of prewarmed sterile saline immediately after surgery for fluid resuscitation. The mice were subcutaneously administered 12.5 mg/kg tramadol for analgesia 30 min before the CLP operation.^30^ A postoperative clinical assessment was performed every 6 h using a modified version of the murine sepsis score.^31^, ^32^ Postoperative monitoring and humane endpoint criteria were established according to Queen's University Standard Operating Procedure 7.32 (Humane Endpoints in Mice with Cecal Ligation and Puncture‐Induced Sepsis). Euthanasia was conducted using sodium pentobarbital at a dose of 150 mg/kg in mice 12 h after CLP surgery, and the hearts were perfused with PBS to eliminate blood impurities. After that, samples of heart tissue were gathered and kept. Mice in the survival study were monitored for 72 h following CLP surgery.

### 2.4 Echocardiography

Twelve hours after the intraperitoneal injection of LPS or CLP surgery, a Vevo 2100 small animal ultrasound device (FUJIFILM VisualSonics Inc., Toronto, Ontario, Canada) was used to scan the experimental mice, and cardiac systolic and diastolic function were measured as previously described.^33^, ^34^ For anaesthesia, mice received isoflurane (3–4% for induction and 1–2% for maintenance) vaporised in 100% oxygen, delivered at 1.0 L/min through a nose cone, and they were positioned supine on a temperature‐controlled heating pad (maintained at 37.0 ± .5°C). Heart rate was continuously monitored via integrated electrocardiographyleads and maintained at approximately 450 beats per minute by adjusting the isoflurane concentration as needed, after which the anterior chest hair was removed. Two‐dimensional and M‐mode transthoracic echocardiography were subsequently employed to evaluate myocardial wall motion, cardiac chamber dimensions and wall thickness, as well as to calculate the ejection fraction (EF) and fractional shortening (FS). Pulsed‐wave Doppler was utilised to determine the peak velocities of mitral inflow, including both the E and A velocities. The isovolumic relaxation time (IVRT) was assessed at the intersection of the left ventricular inflow and outflow tracts of the left ventricle, recorded from an apical five‐chamber view. The time interval of IVRT from the termination of aortic flow to the initiation of mitral inflow was measured.

### 2.5 Western blotting

Protein lysates were prepared from freshly collected myocardial specimens and cultured cells in RIPA lysis buffer enriched with cocktails of protease and phosphatase inhibitors. The BCA method was employed to quantify protein levels in the samples. Next, protein samples were fractionated by 10% SDS‐PAGE and subsequently electroblotted onto PVDF membranes. After blocking the membranes for 2 h at room temperature with 5% non‐fat milk dissolved in TBST, the primary antibodies were incubated for an entire night at 4°C. After three TBST rinses to wash away residual primary antibodies, membranes were exposed to secondary antibodies (#KGC6202‐0.1; KeyGen, Nanjing, Jiangsu, China; #BL004A; Biosharp, Shanghai, China) for 1 h at ambient temperature, with the antibodies suspended in TBST containing 5% non‐fat milk. Protein bands were visualised using an enhanced chemiluminescence reagent (#E423‐01/02; Vazyme, Nanjing, Jiangsu, China). A densitometric analysis was performed using ImageJ v1.42q. Following background removal, each band's integrated density was calculated. Following membrane stripping and reprobing, phosphorylation signals for phospho‐proteins were expressed relative to the abundance of the corresponding total proteins. A researcher who was blinded to the experimental grouping carried out the quantification, and *n* = 6 independent biological replicates per experiment were performed. The antibodies used included the following: Serpina3n (#AF4709; R&D Systems, Minneapolis, Minnesota, USA), Janus kinase (JAK)1 (phospho‐Tyr1034/1035) (#74129; Cell Signaling Technology, Danvers, Massachusetts, USA), JAK1 (#3344; Cell Signaling Technology), signal transducer and activator of transcription (Stat)3 (phospho‐Tyr705) (#9145; Cell Signaling Technology), Stat3 (#10253‐2‐AP; Proteintech, Wuhan, Hubei, China), GAPDH (#A19056; Abclonal, Wuhan, Hubei, China), Lamin B1 (#66095‐1‐Ig; Proteintech), Flag (#20543‐1‐AP; Proteintech) and His‐Tag (#66005‐1‐Ig; Proteintech).

### 2.6 Quantitative real‐time polymerase chain reaction

RNA extraction from the harvested samples was carried out with FreeZol reagent (#R711‐01; Vazyme). Total RNA was converted into complementary DNA (cDNA) using a commercial Kit (#RT101‐01; Vazyme); target transcripts were subsequently measured by quantitative real‐time polymerase chain reaction (qPCR). Using 18s as the reference gene for normalisation, the 2^‐ΔΔCT technique was employed to assess the levels of gene expression. Primer sets used here are provided in Table S1.

### 2.7 Enzyme‐linked immunosorbent assay

Serpina3n concentrations within culture supernatants were determined with a commercial mouse Serpina3n (serine protease inhibitor A3N) enzyme‐linked immunosorbent assay (ELISA) kit (#EM0503; FineTest, Wuhan, Hubei, China), following the manufacturer's guidelines. Readings of optical density (OD) were obtained on a microplate reader (#DTX880; Beckman Coulter, Brea, California, USA). Using a standard curve created with known concentrations of recombinant Serpina3n standards, we calculated the concentration of Serpina3n in each sample by interpolation. Each specimen was assayed in duplicate, and statistical analyses were conducted based on averaged values. Results were expressed in ng/mL.

### 2.8 Immunofluorescence staining

Heart samples were fixed in 4% paraformaldehyde, embedded in paraffin and sliced into 5‐µm sections. Following antigen retrieval in citrate buffer, tissue sections were incubated in 5% BSA for blocking and then treated with  .1% Triton X‐100 for permeabilisation. Following incubation with primary antibodies overnight at 4°C, the tissue samples were exposed to secondary antibodies. DAPI served as the nuclear counterstain. A fluorescent microscope was used to acquire the images. The antibodies used included: Serpina3n (diluted 1:50; #AF4709; R&D Systems), vimentin (diluted 1:50; #A19607; Abclonal), Col1a1 (diluted 1:400; #67288‐1‐Ig; Proteintech), α‐actinin (diluted 1:500; #GB111556; Servicebio, Wuhan, Hubei, China), CD68 (diluted 1:50; #ab53444; Abcam, Cambridge, UK), CD31 (diluted 1:100; #ab222783; Abcam) and Ly6G (diluted 1:400; #A22270; Abclonal).

### 2.9 Immunohistochemical staining

Immunohistochemistry was performed to assess inflammatory cell infiltration. Tissue sections (2 µm thick) were deparaffinised and incubated with 3% H_2_O_2_ and blocking serum, followed by an overnight incubation with an anti‐CD11b recombinant monoclonal antibody (diluted 1:32000; #87419‐1‐RR; Proteintech) and anti‐Ly6G rabbit mAb (diluted 1:400; #A22270; Abclonal). The extent of cardiac inflammatory cell infiltration was quantified in 3 randomly selected high‐power fields (400×) per section from three sections per heart (*n* = 6 mice per group) in a blinded manner. Two researchers who were blind to the groups used ImageJ to manually count, and following uniform threshold settings, the proportion of positive staining area was determined.

### 2.10 Neonatal mouse primary fibroblast and cardiomyocyte isolation

Primary neonatal mouse cardiac fibroblasts (NMCFs) and cardiomyocytes (NMCMs) were harvested from hearts of 1–2‐day‐old C57BL/6 neonatal pups. The hearts were washed in PBS containing heparin sodium and then minced and digested with collagenase and trypsin until the tissue almost completely disappeared. NMCMs were obtained by the differential adhesion method: after 2 h, the nonadherent cell population was harvested and identified as NMCMs. NMCFs and NMCMs were cultivated in DMEM supplemented with a 1% penicillin–streptomycin combination and 10% foetal bovine serum. Immunofluorescence staining was performed to verify the purity of the isolated NMCFs and NMCMs, as shown in Figure S1.

### 2.11 Neonatal mouse primary cardiac endothelial cell isolation

Primary cardiac endothelial cells (NMECs) were isolated from the hearts of neonatal mice as described previously.^35^, ^36^ Hearts were excised, cleared of residual blood in ice‐cold PBS and finely chopped, followed by enzymatic digestion for 25 min at 37°C in DMEM containing collagenase II, collagenase IV and 7.5 µg/mL DNase I. Suspended cells were passed through a 70 µm nylon mesh filter to eliminate tissue fragments and cell clumps, followed by centrifugation at 300×*g* for 5 min. The cells were sequentially labelled with biotinylated anti‐CD31 antibody (#13‐0311‐82; Invitrogen (Thermo Fisher Scientific), Waltham, Massachusetts, USA) and antibiotin MicroBeads (#130‐090‐485; Miltenyi, Bergisch Gladbach, Germany) for 30 min each at 4°C. Cells bearing magnetic labels were applied to LS columns (#130‐042‐401; Miltenyi) mounted within a MidiMACS™ Separator (#130‐042‐302; Miltenyi). Following three rinsing steps, the columns were taken out of the magnetic field, and enriched cells were eluted using culture medium. Purified NMECs were cultured in endothelial cell (EC) growth medium (#1001; ScienCell Research Laboratories, Carlsbad, California, USA).

### 2.12 Adult mouse CF, CM and EC isolation

Adult mouse CFs, adult mouse CMs and ECs were isolated as previously reported.^37^, ^38^, ^39^ After adult mice were put to sleep with 4% isoflurane, their hearts were excised immediately and mounted onto the Langendorff perfusion system. Tyrode's solution without calcium was initially used to perfuse the hearts for 5 min, followed by perfusion with a digestion mixture containing trypsin, type II collagenase and protease. The left ventricle was isolated, minced in an enzyme solution supplemented with  .2% BSA and filtered through a 100 µm cell strainer. The filtrate was allowed to stand at 22°C for 10 min to allow the gravity sedimentation of CMs. The supernatant obtained after gravity settling was centrifuged at 250×*g* for 10 min to pellet the CFs, which were then re‑suspended in fibroblast growth medium, plated onto culture dishes and thoroughly washed 2 h after plating to eliminate nonadherent cells.

As guided by the manufacturer's protocol, nonadherent cells from the fibroblast plating stage were collected for MACS using anti‐CD31 magnetic microbeads (#130‐097‐418; Miltenyi). The cell‐bead suspension was loaded onto LS columns (#130‐042‐401; Miltenyi). To eliminate unlabelled cells, the column was washed three times with MACS buffer (DPBS,  .5% BSA, 2 mM EDTA). CD31‐positive ECs were collected and eluted with culture medium after the column was removed from the magnetic field. Enriched EC populations were maintained using endothelial growth medium (#1001; ScienCell).

### 2.13 Treatment with the recombinant Serpina3n protein

The recombinant mouse Serpina3n protein (r‐Serpina3n; #4709‐PI; R&D Systems) was used for in vitro experiments. The protein was produced within a mouse myeloma cell line (NS0‐derived), representing the Phe21–Lys418 fragment fused to a C‐terminal 6×His tag; its theoretical molecular weight is 46 kDa. The purity was >85%, as determined by SDS‐PAGE under reducing conditions and visualisation by silver staining. Endotoxin content was below 1.0 EU for each microgram of protein, quantified via the LAL assay. The protein was supplied as a solution in Tris and NaCl that underwent sterilisation via a  .2 µm filter membrane and preserved at −80°C prior to experimentation. For cellular treatments, the protein was diluted in sterile PBS to the indicated final concentrations. The working concentration (100 ng/mL) was selected based on a previous study using the same recombinant mouse Serpina3n protein.^40^

### 2.14 Adenovirus infection and siRNA transfection

To achieve Serpina3n overexpression in NMCFs, the cells were infected with an adenovirus carrying the Serpina3n gene (Ad‐Serpina3n; Genechem, Shanghai, China) for 8 h, with an empty vector adenovirus (Ad‐null) serving as the control. A Western blot (WB) analysis of Serpina3n protein levels was used to confirm the overexpression effectiveness. For endogenous Serpina3n knockdown, NMCFs received small interfering RNA (siRNA) directed against Serpina3n (Si‐Serpina3n, 50 nmol/L; Zebrafish Biotech, Nanjing, Jiangsu, China). Transfection was carried out employing Lipofectamine 3000 transfection reagent (#L3000015; Invitrogen) according to the instructions supplied by the manufacturer, with a scrambled siRNA (siNC) used as the negative control. Total protein was extracted, and the knockdown efficiency was confirmed by WB. The target siRNA sequences are detailed in Table S2.

### 2.15 Plasmid transfection

After being cloned, the Stat3 overexpression plasmid was added to the pcDNA3.1 vector. As directed by the manufacturer, NMCFs were transfected with the Stat3 overexpression plasmid (Genechem) using Lipofectamine 3000 reagent (#L3000015; Invitrogen). LPS was used to activate the cells 48 h after transfection.

### 2.16 Indirect coculture model

NMCFs were incubated with Serpina3n‐overexpressing adenovirus for 8 h or with Serpina3n‐knockdown siRNA for 6 h. Forty‐eight hours after transfection, conditioned medium from transfected NMCFs was harvested and transferred to NMCM cultures.

### 2.17 Measurement of lactate dehydrogenase levels

After NMCMs were treated with LPS or r‐Serpina3n for 12 h, culture supernatants were collected to measure lactate dehydrogenase (LDH) levels. Commercial assay kits (#A020‐2; Nanjing Jiancheng Bioengineering Institute, Jiangsu, China) were utilised for LDH detection following the protocols supplied by the manufacturer. OD values at 440 nm were acquired with a multimode microplate reader (#DTX880; Beckman Coulter).

### 2.18 Cell viability assay

NMCMs were plated onto 96‐well plates, with six replicate wells assigned to each experimental group. Following 12 h treatment of NMCMs with LPS, r‐Serpina3n, S3I‐201 or Colivelin, the cultures were exposed to 10% CCK‐8 reagent (#KGA9310; KeyGen) and kept for 2 h at 37°C in darkness. Absorbance values were recorded via a multi‐function microplate reader (#DTX880; Beckman Coulter).

### 2.19 Chromatin immunoprecipitation assays

A ChIP Assay Kit (#P2078, Beyotime, Haimen, Jiangsu, China) was used to conduct chromatin immunoprecipitation (ChIP) tests. Briefly, the NIH3T3 cells were seeded and cultured to the appropriate density. The cells were harvested and repeatedly rinsed with PBS containing 1 mM PMSF after being cross‐linked with 1% formaldehyde at 37°C for 10 min and quenched with glycine. They were then counted and divided into tubes containing 1 × 10^6^ cells per tube. Cells were disrupted in SDS lysis buffer spiked with 1 mM PMSF during 10 min of incubation on ice. Sonication was used to shear the genomic DNA into 200–1000 bp pieces (2 mm probe, 3 s on/3 s off, 30 cycles). The supernatant obtained by centrifugation was diluted, with 20 µL reserved as the input. Nonspecific binding was blocked with Protein A+G Agarose/salmon sperm DNA in the remaining sample, which was then split equally into two tubes: overnight incubation at 4°C was performed for one sample with anti‑Flag antibody and another with an isotype IgG control. Protein A+G Agarose was used to precipitate immune complexes, which were then successively cleaned using low‐salt, high‐salt, LiCl and TE buffers. Finally, 5 µL of the precipitate was treated with lysis buffer and incubated at 65°C and 98°C, the supernatant was collected and qPCR was performed. The primer sequences for all the predicted binding sites are listed in Table S3.

### 2.20 Dual‐luciferase reporter assay

Following treatment, dual‐luciferase reporter experiments were performed on the 293T cell line with the Dual‐Lumi™ Luciferase Reporter Gene Assay Kit (#RG088S; Beyotime) strictly according to the operational guidelines provided by the manufacturer. After discarding the culture supernatant, the cell monolayers underwent a single PBS wash. Each culture well was then filled with 200 µL of lysis buffer designed for reporter gene detection. Samples were then left at ambient temperature for 10 min with repeated pipetting to ensure total cellular lysis. After centrifugation of the cell lysate at 9000 ×g for 5 min, the supernatant was transferred to new microcentrifuge tubes. For each sample, 100‐µL aliquot of firefly luciferase detection solution was added to 20 µL of cell lysate, followed by incubation for 10 min, after which the luminescence intensity was measured. After adding the renilla luciferase test reagent, the mixture was vigorously mixed and incubated for 10 more minutes. The luminescence intensity was then measured once again.

### 2.21 RNA sequencing analysis

We isolated total RNA from the collected cardiac tissues of fibroblast‐specific Serpina3n KO mice and their littermate controls with SIMD using a Freezol reagent kit (#R711‐01; Vazyme). Three independent biological replicates were harvested for every experimental group. RNA quality was assessed with a 2100 Bioanalyzer, which verified that the purity and integrity of the extracted RNA met the experimental requirements. Novogene Co., Ltd. (Beijing, China) received the qualifying RNA samples for an RNA‐seq study. Differential expression analysis was performed with R (v4.4.2). Genes categorised as differentially expressed (DEGs) met thresholds of FDR‐adjusted *p* values < .05 and absolute log2 fold change > 1. Functional enrichment for these DEGs was interpreted via Kyoto Encyclopedia of Genes and Genomes (KEGG) and Gene Set Enrichment Analysis (GSEA) pathway analysis.

### 2.22 GEO database

Detailed information is provided in the Supporting Information.

### 2.23 Data analysis

GraphPad Prism 10.2.1 (GraphPad Software Inc., La Jolla, California, USA) was employed to conduct statistical evaluations. All data were presented as the mean ± standard deviation (SD), with *n* representing the number of biological replicates. Biological replicates were defined as independent cell culture preparations or individual mice. Normality and homogeneity of variance were assessed prior to parametric testing. Normality was assessed with the Shapiro–Wilk test, while homogeneity of variances was evaluated using the Brown–Forsythe test. When the data met the assumptions of a normal distribution and equal variance, parametric tests were applied. An unpaired two‐tailed Student's *t*‐test was applied for comparisons between two separate experimental cohorts. One‐way analysis of variance (ANOVA) was adopted when analysing three or more groups, with Tukey's post hoc test implemented for pairwise multiple comparisons. For experiments involving two independent categorical variables, two‐way ANOVA was performed, followed by Šidák‐corrected post hoc comparisons for selected pairwise group comparisons. Nonparametric tests (Kruskal–Wallis test for multiple groups; Mann–Whitney *U* test for two groups) were used when parametric assumptions were broken. Kaplan–Meier curves were created for the survival analysis, and the log‐rank (Mantel–Cox) test was used to assess group differences. Corrections for multiple comparisons were applied as indicated above (Tukey's test for one‐way ANOVA; Šidák test for two‐way ANOVA post hoc comparisons). Using a computer‐generated random number sequence, the mice were divided into experimental groups at random. All animal treatments, data collection and outcome assessments were performed by researchers who were blinded to the group allocation. For in vitro experiments, cell culture plates were randomly allocated to treatment conditions, and both sample collection and data processing were conducted blindly using anonymised sample codes.

## 3 RESULTS

### 3.1 Serpina3n expression is up‐regulated in mice with SIMD and primarily originates from fibroblasts

To explore the association between Serpina3n and SIMD, we analysed Serpina3n mRNA expression in heart tissues from mice with and without LPS stimulation using the GSE267388 and GSE215955 datasets. Serpina3n mRNA expression was observed to be markedly elevated in the hearts of sepsis‐affected animals (Figure 1A–C). Next, we analysed the GSE229925 dataset retrieved from the GEO database, and the findings showed that Serpina3n expression was significantly elevated in mice with CLP‐induced SIMD (Figure 1D). Among the Serpina3 family members, Serpina3n was most prominently up‐regulated in septic heart tissue, while the expression of the other family members did not increase significantly (Figure 1E). We subsequently established a mouse model of SIMD via intraperitoneal injection of LPS or CLP surgery. In the mouse SIMD models produced by LPS and CLP, both WB and qPCR verified that Serpina3n protein and mRNA levels were markedly elevated (Figure 1F,G). We first evaluated alterations in cardiac cell populations between mice with and without sepsis by identifying cell types based on scRNA‐seq data and detected 13 distinct cell clusters and their proportional distributions (Figure 1H,I). Moreover, we conducted an annotation analysis using the markers of each cell (Figure S2A). We further defined the cellular source of Serpina3n in cardiac tissue by analysing single‐cell data and observed that Serpina3n expression strongly overlapped with the expression of genes serving as markers of CFs (Figure 1J). In addition, a violin plot revealed that Serpina3n was expressed mainly in fibroblasts and that its expression in fibroblasts was markedly elevated in the SIMD model mice (Figure S2B,C). We subsequently performed a cell annotation analysis using the GSE285720 database to further verify these results. The level of Serpina3n was augmented in SIMD mice and the increase was significant in activated fibroblasts (Figure S3A,B). In this database, we identified 11 different cell clusters and their proportions (Figure S3C,D). Moreover, we performed an annotation analysis of the marker genes expressed in each cell in this database (Figure S3E). Furthermore, the results from this database also revealed that the expression of the Serpina3n gene strongly overlapped with the expression of fibroblast marker genes (Figure S3F). Immunofluorescence staining revealed increased Serpina3n expression in SIMD mice, and Serpina3n was colocalised mainly with the fibroblast markers vimentin and Col1a1, but not with cardiomyocyte (CM) (α‐actinin), endothelial (CD31), macrophage (CD68) or neutrophil (Ly6G) markers (Figures 1K and S4A). Next, we explored the optimal concentration and duration of LPS treatment in NMCFs and found that Serpina3n protein expression was most significantly induced in NMCFs following treatment with 1 or 10 µg/mL of LPS for 12 h (Figure 1L). Given that CMs and CFs are the major cell types in the heart, we examined Serpina3n expression in these two cell types under SIMD conditions. NMCMs and NMCFs were stimulated with 1 µg/mL LPS for 12 h. WB analysis revealed that the administration of LPS clearly increased the Serpina3n protein levels in NMCFs but not in NMCMs (Figure 1M). Furthermore, we detected the levels of secreted Serpina3n in the supernatants of LPS‐treated NMCFs, NMCMs and NMECs. The results of the ELISA also confirmed that NMCFs are the predominant cellular source of Serpina3n (Figure S4B). More importantly, Serpina3n protein expression was increased in NMCMs upon co‐culture with NMCFs, while Serpina3n mRNA levels remained unchanged (Figure S4C). All of these findings suggest that Serpina3n is up‐regulated in the setting of SIMD and predominantly enriched in CFs rather than other cells.

**FIGURE 1:**
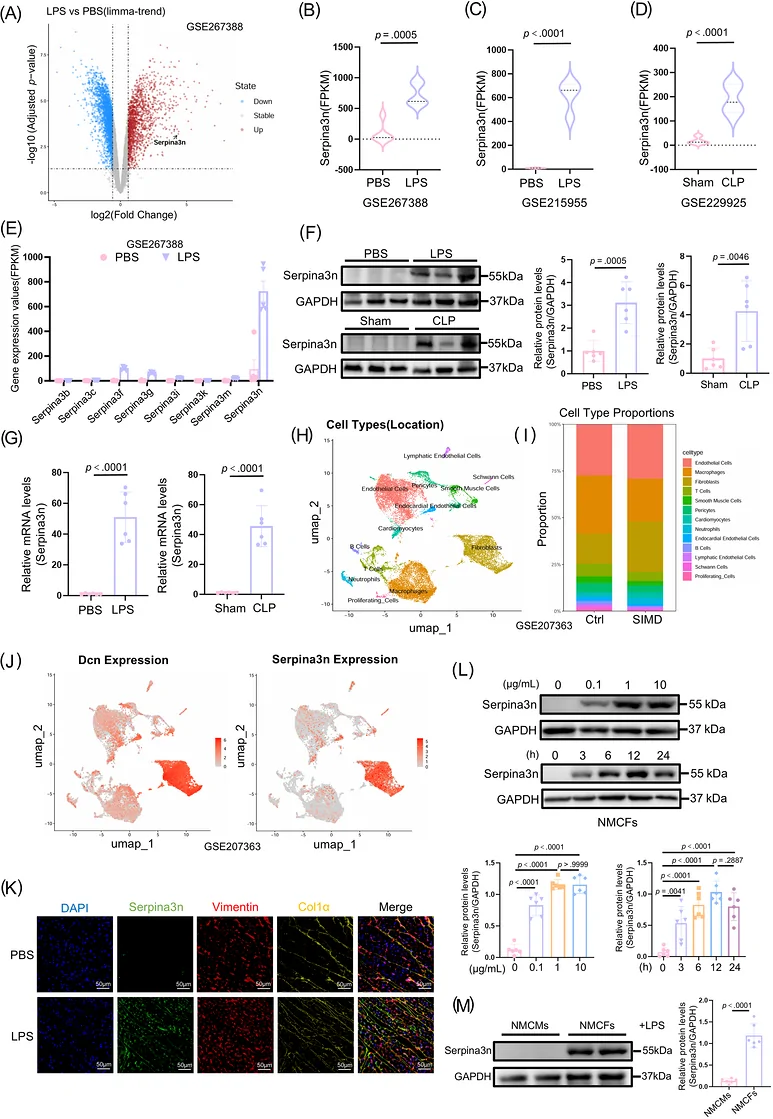
Serpina3n expression is markedly up‐regulated in the hearts of mice with sepsis and is primarily derived from fibroblasts. (A–D) An analysis of the GEO database revealed increased expression of Serpina3n in the cardiac tissue of mice treated with LPS or mice with caecal ligation and puncture (CLP)‐induced SIMD. (E) Among the Serpina3 family members detected in mice with SIMD, the expression of Serpina3n in the heart was the most significantly increased. (F) Expression of the Serpina3n protein in cardiac tissue after an intraperitoneal LPS injection and CLP. (G) Expression of the Serpina3n mRNA in cardiac tissue after an intraperitoneal LPS injection and CLP. (H) UMAP plot showing the presence of 13 distinct cardiac cell types. (I) The percentage of cells in mice with or without SIMD. (J) Colocalisation of the expression of the Serpina3n gene with the expression of fibroblast marker genes. (K) Immunofluorescence staining and co‐localisation of Serpina3n in cardiac tissues from wild‐type (WT) mice and mice with sepsis. Scale bars: 50 µm. (L) Expression of the Serpina3n protein after treatment with different concentrations of LPS for different durations. (M) Abundance of the Serpina3n protein in NMCMs and NMCFs after LPS stimulation. Densitometric quantification of the Western blots in (F), (L and M) was performed using ImageJ, with GAPDH used as a loading control. The data are shown as the mean ± SD. Data shown in (F and G) represent *n* = 6 mice per group, and data in (L and M) represent *n* = 6 independent biological replicates. Statistical significance was determined using one‐way ANOVA followed by Tukey's post hoc test for the quantitative analyses shown in (B–D), (F), (G), (L) and (M). For the analysis of the data in (E), two‐way ANOVA was applied. Public RNA‐seq data GSE267388, GSE215955, GSE229925 and GSE207363 were retrieved from NCBI GEO database. *Abbreviation*: CLP, caecal ligation and puncture.

### 3.2 Serpina3n KO exacerbates cardiac function and inflammation in mice with LPS‐induced SIMD

We investigated the role of Serpina3n in SIMD by generating Serpina3n KO mice using CRISPR/Cas9‐mediated deletion of the sequence between exon 1 and exon 2 of the Serpina3n gene. The complete ablation of Serpina3n in cardiac tissue was confirmed by WB (Figure 2A). Sepsis was then induced in 6–8‐week‐old WT and Serpina3n KO mice via an intraperitoneal LPS injection. We first observed that the survival rate was significantly lower in septic Serpina3n KO mice than in septic WT mice (Figure 2B). Echocardiography was used to evaluate cardiac function 12 h following LPS stimulation. Cardiac function was decreased in both groups of mice with sepsis compared with control mice, and cardiac systolic function was significantly worse in Serpina3n KO mice than in WT mice, as reflected by the EF and FS values, whereas no significant difference in cardiac diastolic function was observed (Figure 2C–G). We next examined the expression of pro‐inflammatory cytokines in mouse cardiac tissue. qPCR revealed that the mRNA levels of pro‐inflammatory cytokines were higher in the hearts of Serpina3n KO mice with sepsis than in those of WT mice with sepsis (Figure 2H–J). Furthermore, a histopathological analysis via immunohistochemical staining revealed that inflammatory cell infiltration in the heart tissue of Serpina3n KO mice with sepsis was substantially elevated compared with WT mice with sepsis (Figure 2K). Moreover, we established a CLP‐induced sepsis model using WT and Serpina3n KO mice. Compared with WT control mice, Serpina3n KO mice exhibited lower survival rates, deteriorated cardiac function and aggravated myocardial inflammatory cell infiltration (Figure S5). Collectively, these results reveal that Serpina3n deficiency exacerbates both cardiac dysfunction and inflammatory cell infiltration in the heart in SIMD.

**FIGURE 2:**
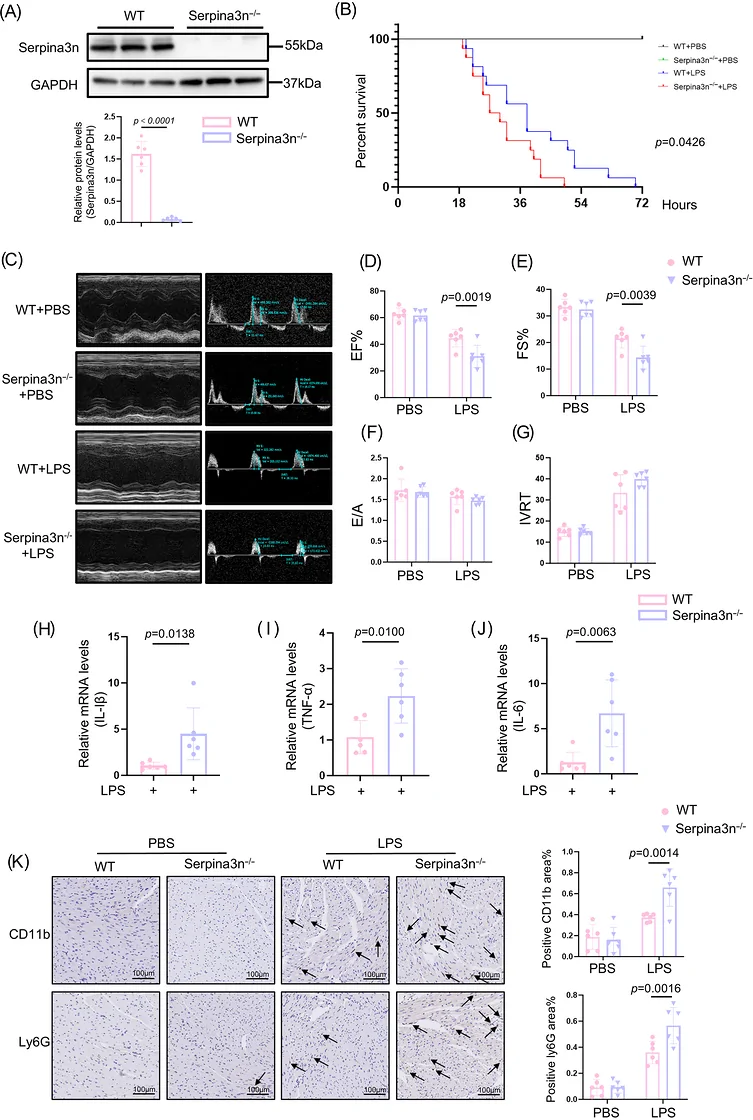
The loss of Serpina3n deteriorates cardiac function and inflammation in the hearts of mice with LPS‐induced sepsis. (A) Abundance of the Serpina3n protein in cardiac tissues from WT and Serpina3n^−/−^ mice. (B) Survival curves of WT and Serpina3n^−/−^ mice injected with or without LPS. (C) Representative echocardiograms of WT and Serpina3n^−/−^ mice at 12 h after LPS administration. (D–G) The ejection fraction (EF), fractional shortening (FS), E/A ratio and isovolumic relaxation time (IVRT) were quantified in the columns. (H–J) Relative mRNA levels of inflammatory factors in the cardiac tissues of WT and Serpina3n^−/−^ mice after LPS administration. (K) Representative images of CD11b and Ly6G staining in cardiac tissue sections from the experimental mice. Scale bars: 100 µm. Densitometric quantification of the Western blots in (A) was performed using ImageJ, with GAPDH serving as a loading control. All the data are shown as the mean ± SD. Data shown in (A and D–K) represent *n* = 6 mice per group, and data in (B) represent *n* = 16 mice per group. Statistical analyses of the data presented in (A), and (H–J) were performed using one‐way ANOVA with Tukey's post hoc test. For the analysis of the data in (D–G), and (K), two‐way ANOVA was applied.

### 3.3 Fibroblast‐specific Serpina3n deficiency exacerbates myocardial function and inflammation in mice with LPS‐induced SIMD

Given that Serpina3n expression was significantly increased in cardiac tissues in the SIMD model mice and that Serpina3n was predominantly localised in mouse CFs but not in other cells, we constructed fibroblast‐specific Serpina3n KO (Col1a2‐iCre) mice and their littermate control (Serpina3n^flox/flox^) mice using Cre‐flox technology. WB analysis confirmed Serpina3n deletion in CFs of Col1a2‐iCre mice and validated that Serpina3n is selectively knocked out in CFs within the heart (Figures 3A and S6). Following an intraperitoneal LPS injection to induce sepsis in 6–8‐week‐old Serpina3n^flox/flox^ and Serpina3n Col1a2‐iCre mice, we observed a significantly lower survival rate in Serpina3n Col1a2‐iCre mice with sepsis than in their littermate controls with sepsis (Figure 3B). Echocardiographic assessment revealed significant cardiac dysfunction in both groups. Moreover, compared with those of flox/flox mice, the EF and FS of Col1a2‐iCre mice were significantly worse, whereas no notable difference in diastolic function was observed between the two experimental groups (Figure 3C–G). Consistently, the results of the qPCR analyses revealed higher expression levels of pro‐inflammatory cytokines in the cardiac tissue of Col1a2‐iCre mice than in that of flox/flox control mice (Figure 3H–J). Additionally, histopathological analysis via immunohistochemical staining revealed markedly increased inflammatory cell infiltration in the cardiac tissues of Col1a2‐iCre mice with SIMD compared with that in the cardiac tissues of flox/flox mice with SIMD (Figure 3K). Furthermore, we constructed a CLP‐induced sepsis model using 6–8‐week‐old fibroblast‐specific Serpina3n KO mice and their littermate control mice. Notably, compared with littermate controls, mice with a fibroblast‐specific Serpina3n deletion had significantly reduced survival rates, impaired cardiac function and exacerbated myocardial inflammatory cell infiltration (Figure S7). In summary, these data indicate that fibroblast‐specific deficiency of Serpina3n exacerbates both cardiac dysfunction and myocardial inflammatory cell infiltration in mice with SIMD.

**FIGURE 3:**
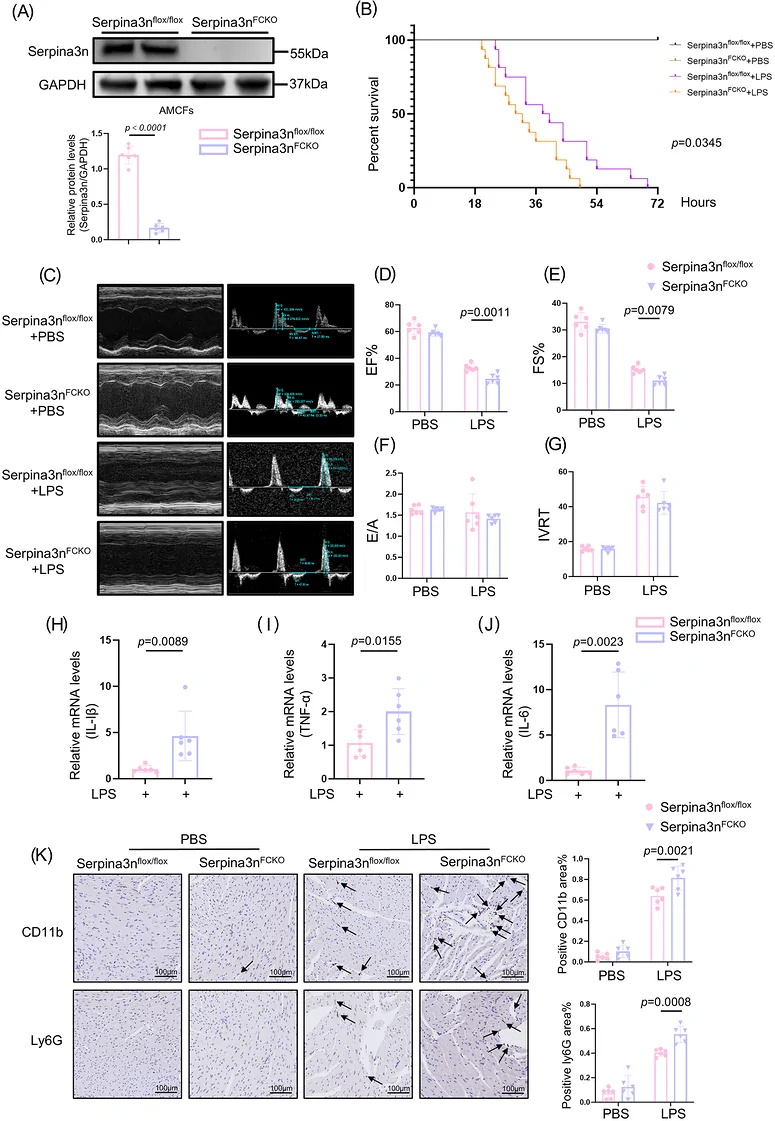
Fibroblast‐specific Serpina3n deficiency induces cardiac dysfunction and inflammation in the hearts of mice with LPS‐induced sepsis. (A) Abundance of the Serpina3n protein in cardiac fibroblasts from flox/flox and Serpina3n Col1a2‐iCre mice. (B) Survival curves of flox/flox and Serpina3n Col1a2‐iCre mice injected with or without LPS. (C) Representative echocardiograms of flox/flox and Serpina3n Col1a2‐iCre mice at 12 h after LPS administration. (D–G) The ejection fraction (EF), fractional shortening (FS), E/A ratio and isovolumic relaxation time (IVRT) were quantified in the columns. (H–J) Relative mRNA levels of inflammatory factors in the cardiac tissues of flox/flox and Serpina3n Col1a2‐iCre mice after LPS administration. (K) Representative images of CD11b and Ly6G staining in cardiac tissue sections from the experimental mice. Scale bars: 100 µm. Densitometric quantification of the Western blots in (A) was performed using ImageJ, with GAPDH serving as a loading control. All the data are shown as the mean ± SD. Data shown in (A and D–K) represent *n* = 6 mice per group, and data in (B) represent *n* = 16 mice per group. Statistical analyses of the data presented in (A), and (H–J) were performed using one‐way ANOVA with Tukey's post hoc test. For the analysis of the data in (D–G) and (K), two‐way ANOVA was applied. *Abbreviations*: FCKO: fibroblast‐specific Serpina3n KO; AMCFs, adult mouse cardiac fibroblasts.

### 3.4 Serpina3n from CFs mediates CF‒CM crosstalk and ameliorates LPS‐induced SIMD

CMs express Serpina3n at very low levels (Figure 1M). Single‐cell CellChat analysis revealed increased communication between fibroblasts and CMs, reflected in both the number and the strength of cell–cell interactions (Figure 4A). In addition, NMCMs can take up exogenous Serpina3n, as shown by the intracellular Flag signal after incubation with conditioned medium from Flag tagged Serpina3n‐overexpressing NMCFs and by the uptake of recombinant His tagged Serpina3n (Figure S8A). On the basis of these observations, we reasoned that CF‐derived Serpina3n might promote CF–CM crosstalk and exert cardioprotective effects in SIMD. We validated this hypothesis by first isolating primary NMCMs. One group of NMCMs was directly stimulated with LPS, while the other group was treated with recombinant Serpina3n protein (r‑Serpina3n) at the time of LPS stimulation, with the goal of determining the functional role of Serpina3n in LPS‐induced inflammation (Figure 4B). According to the experimental data, r‐Serpina3n markedly suppressed the mRNA expression of TNF‐α, IL‐6 and IL‐1β in NMCMs (Figure 4I–K). In addition, r‐Serpina3n rescued LPS‐mediated decreases in cell viability and attenuated LDH release (Figure S8B).

**FIGURE 4:**
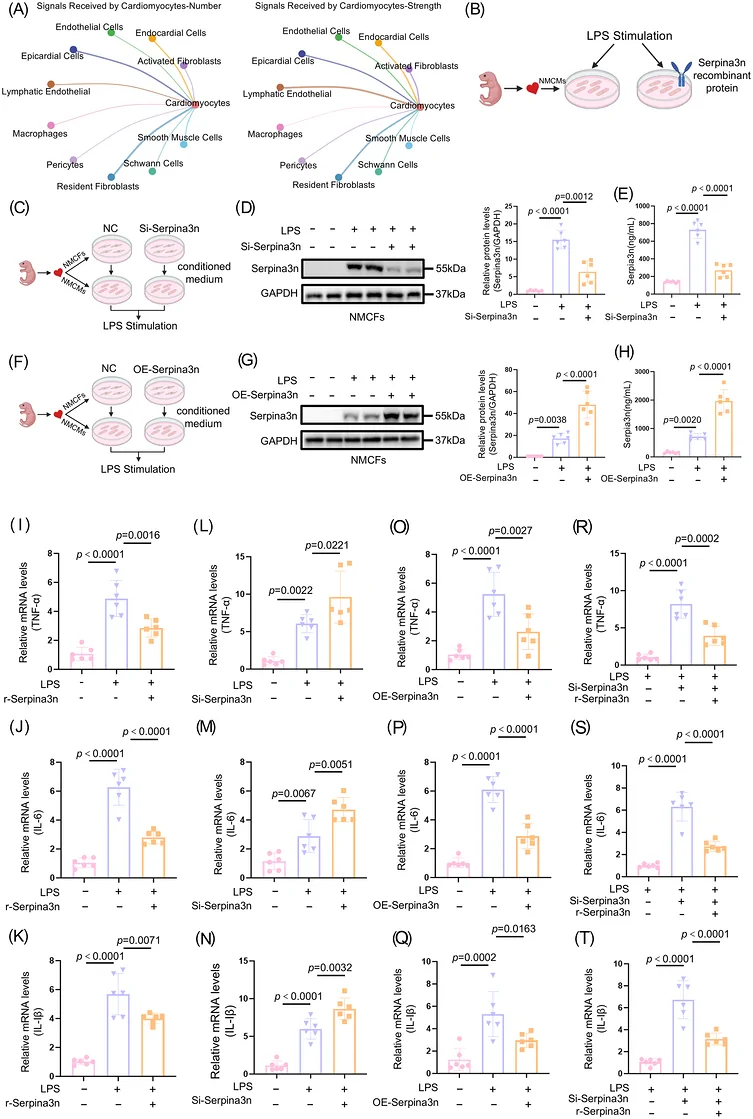
Cardiac fibroblast‐derived Serpina3n is involved in fibroblast–cardiomyocyte crosstalk and ameliorates LPS‐induced SIMD. (A) Single‐cell CellChat analysis revealed an increased number of cell–cell communications and enhanced interaction strength between fibroblasts and cardiomyocytes. Public single‐cell RNA‐seq data GSE285720 was downloaded from NCBI GEO database. (B) Experimental design. One group of NMCMs was directly subjected to LPS, whereas the other group received LPS together with r‐Serpina3n. (C) Experimental design. Serpina3n was silenced with an siRNA in NMCFs. The corresponding NMCMs were treated with conditioned medium collected from NMCFs and subsequently stimulated with LPS. (D) Expression of the Serpina3n protein in NMCFs after treatment with the siRNA. (E) Serpina3n protein levels in the conditioned medium of NMCFs after treatment with the siRNA. (F) Experimental design. Adenovirus‐mediated Serpina3n overexpression (OE‐Serpina3n) in NMCFs. The corresponding NMCMs were treated with conditioned medium collected from NMCFs and then subjected to LPS stimulation. (G) Expression of the Serpina3n protein in NMCFs after adenovirus‐mediated overexpression. (H) Serpina3n protein levels in the conditioned medium of NMCFs after adenovirus‐mediated overexpression. (I–K) Relative mRNA expression of the inflammatory factors TNF‐α (I), IL‐6 (J) and IL‐1β (K) in LPS‐stimulated NMCMs treated with or without r‐Serpina3n. (L–N) Relative mRNA expression of the inflammatory factors TNF‐α (L), IL‐6 (M) and IL‐1β (N) in NMCMs cultured with conditioned medium from NMCFs treated with or without Si‐Serpina3n. (O–Q) Relative mRNA expression of the inflammatory factors TNF‐α (O), IL‐6 (P) and IL‐1β (Q) in NMCMs cultured with conditioned medium from NMCFs treated with or without OE‐Serpina3n. (R–T) Relative mRNA expression of the inflammatory factors TNF‐α (R), IL‐6 (S) and IL‐1β (T) in NMCMs cultured with conditioned medium from Si‐Serpina3n‐treated NMCFs with or without r‐Serpina3n supplementation. Densitometric quantification of the Western blots in (D and G) was performed using ImageJ, with GAPDH serving as a loading control. All the data are shown as the mean ± SD. Data shown in (D, E and G–T) represent *n* = 6 independent biological replicates. Statistical analyses of the data presented in (D–E), and (G–T) were performed using one‐way ANOVA with Tukey's post hoc test. *Abbreviations*: NMCFs, primary neonatal mouse cardiac fibroblasts; NMCMs, primary neonatal mouse cardiomyocytes; r‐Serpina3n, recombinant mouse Serpina3n protein; Si‐Serpina3n, Serpina3n small interfering RNA (siRNA); OE‐Serpina3n, Serpina3n overexpression.

To gain deeper insight into how Serpina3n exerts protection under inflammatory conditions, we constructed a Serpina3n‐overexpressing (OE‐Serpina3n) adenovirus and Serpina3n‐knockdown siRNA (Si‐Serpina3n). First, we knocked down Serpina3n in NMCFs using Si‐Serpina3n. After confirming efficient knockdown via WB analysis and measuring Serpina3n levels in NMCF culture supernatants using ELISA, the supernatant from NMCFs was transferred to NMCMs, followed by stimulation with LPS (Figure 4C–E). Si‐Serpina3n dramatically raised the mRNA levels of pro‐inflammatory cytokines in NMCMs as compared with the control treatment (Figure 4L–N). Additionally, we overexpressed Serpina3n in NMCFs via an adenovirus. Following the verification of overexpression via WB analysis and measurement of Serpina3n levels in NMCF culture supernatants using ELISA, the supernatant was transferred to NMCMs, which were then stimulated with LPS (Figure 4F–H). In this experiment, we observed a significant decrease in the mRNA expression of inflammatory markers (Figure 4O–Q). Next, we conducted rescue experiments to validate the protective function of Serpina3n. Briefly, r‐Serpina3n was added to the conditioned medium from Serpina3n‐knockdown NMCFs, and NMCMs were cultured in this conditioned medium to determine whether exogenous Serpina3n could restore the protective phenotype. Our results revealed that the increased mRNA levels of pro‐inflammatory cytokines in NMCMs induced by Si‐Serpina3n were effectively reversed by r‐Serpina3n treatment, thereby revealing that Serpina3n exerts a cardioprotective effect (Figure 4R–T). These results indicate that upon LPS stimulation, Serpina3n secreted by NMCFs alleviates NMCMs inflammation through paracrine signalling.

### 3.5 Stat3 promotes the transcription of Serpina3n

We investigated the molecular mechanism underlying the significant up‐regulation of Serpina3n in fibroblasts during SIMD by analysing databases such as CISBP, HOCOMOCO and JASPAR and constructed a Venn diagram. The results revealed that Bcl6, Spi1, Egr2, Myog, Klf4, Atoh1, Stat3, Rxra, Bhlha15 and Ar may be the transcription factors that regulate Serpina3n expression (Figure 5A). We subsequently validated the expression levels of the candidate transcription factors using the single‐cell database GSE285720. The findings showed that Myog, Atoh1 and Bhlha15 were not effectively expressed in this dataset (Figure S9A). Further evaluation of the activity of each transcription factor via a single‐cell regulatory network analysis revealed that Stat3 exhibited extremely strong transcriptional regulatory activity in activated fibroblasts (Figure 5B). The heatmap also confirmed that Stat3 was among the top 5 transcription factors with the highest activity in this cell population (Figure S9B). In addition, the density plot of Stat3 transcriptional activity indicated that its spatial distribution strongly overlapped with that of the activated fibroblast cluster (Figures 5C and S3C). Based on the results from this multidimensional analysis, we selected Stat3 as the research focus for subsequent experiments. As a method to verify these results, we constructed a Stat3 overexpression plasmid to increase its expression in fibroblasts. The overexpression of the Stat3 gene in NMCFs was verified by qPCR (Figure S10A), and the mRNA expression of Serpina3n in NMCFs was also up‐regulated after Stat3 transfection (Figure S10B). At the protein level, we detected that the levels of p‐Stat3, Stat3 and Serpina3n in NMCFs increased after Stat3 transfection (Figure S10C). After transfection with the Stat3 plasmid, we subsequently stimulated NMCFs with LPS. This treatment up‐regulated the mRNA expression of both Stat3 and Serpina3n (Figure 5D,E) and increased the protein abundance of p‐Stat3, Stat3 and Serpina3n (Figure 5F). These findings indicate that Stat3 is highly likely to be the transcription factor that regulates the function of Serpina3n. We explored the mechanism by which Stat3 activates Serpina3n and confirmed through ChIP‐PCR that Stat3 promotes the transcription of Serpina3n by binding to the promoter region of the Serpina3n gene (Figure 5G). To verify the functional relevance of this binding, a dual‐luciferase reporter experiment was conducted in the 293T cell line. The results revealed that the Stat3 overexpression plasmid clearly increased luciferase activity (Luc/Rluc ratio), leading to the significant activation of the Serpina3n promoter and increased transcription of Serpina3n (Figure 5H). In conclusion, these observations support direct binding of Stat3 to the Serpina3n promoter, leading to enhanced transcriptional activity of this gene in fibroblasts.

**FIGURE 5:**
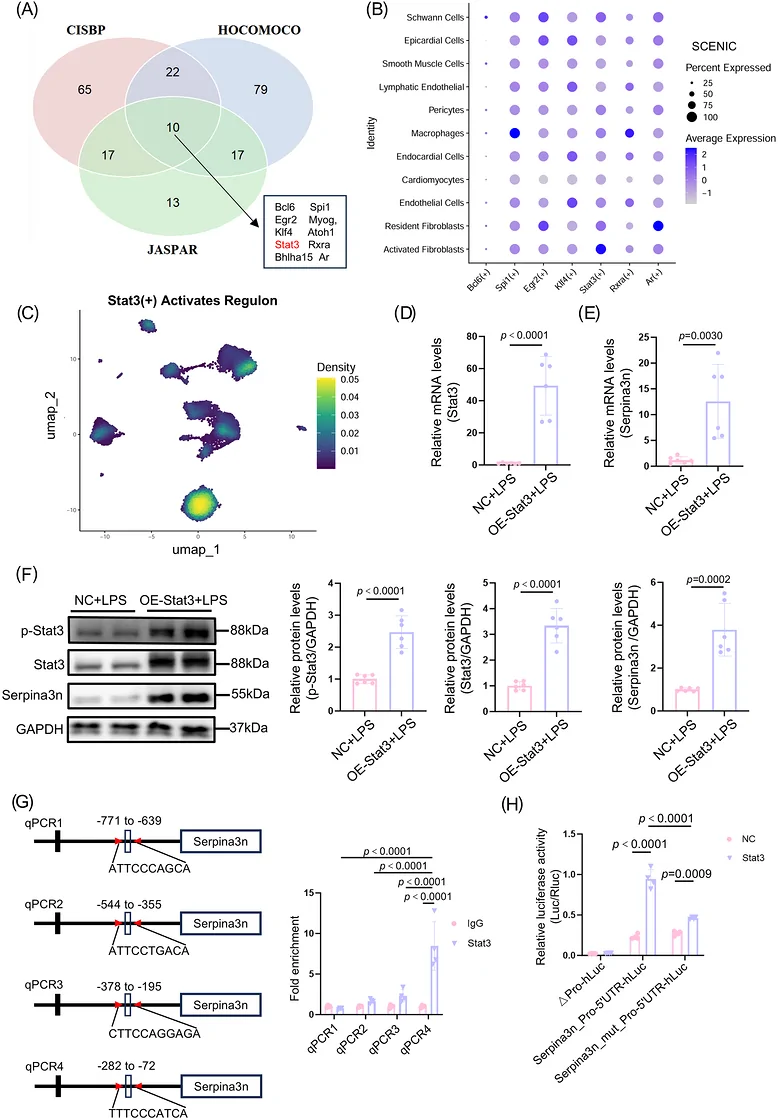
Stat3 facilitates Serpina3n transcription. (A) Venn diagram showing the intersections of the results from the CISBP, HOCOMOCO and JASPAR databases. (B) Dot plot showing the transcriptional activity of various transcription factors in different cell types. Public single‐cell RNA‐seq data GSE285720 was downloaded from NCBI GEO database. (C) Stat3(+) activates Regulon in different cell types. Public single‐cell RNA‐seq data GSE285720 was downloaded from NCBI GEO database. (D) Expression of the Stat3 mRNA in NMCFs after Stat3 plasmid transfection followed by LPS stimulation. (E) Expression of the Serpina3n mRNA in NMCFs after Stat3 plasmid transfection followed by LPS stimulation. (F) Expression of the p‐Stat3, Stat3 and Serpina3n proteins in NMCFs after Stat3 plasmid transfection followed by LPS stimulation. (G) ChIP assays revealed that Stat3 promotes Serpina3n transcription by binding to the Serpina3n promoter. (H) Dual‐luciferase reporter assays indicated that Stat3 binds to the Serpina3n promoter and promotes its transcription. Densitometric quantification of the Western blots in (F) was performed using ImageJ, with GAPDH serving as a loading control. All the data are shown as the mean ± SD. Data shown in (D–F) represent *n* = 6 independent biological replicates, and data in (G and H) represent *n* = 4 independent biological replicates. Statistical analyses of the data presented in (D), (E) and (F) were performed using one‐way ANOVA with Tukey's post hoc test. For the analysis of the data in (G) and (H), two‐way ANOVA was applied. *Abbreviation*: OE‐Stat3, Stat3 overexpression.

### 3.6 Serpina3n mediates CF–CM crosstalk and protects CM by inhibiting the JAK–STAT pathway

We performed a transcriptomic analysis of cardiac tissues from Col1a2‐iCre mice and control littermates after LPS stimulation to dissect molecular mechanisms responsible for Serpina3n‐mediated cardioprotection. The KEGG enrichment analyses revealed that the JAK–STAT pathway ranked as the top enriched pathway in the hearts of Col1a2‐iCre mice with sepsis compared with those of control mice with sepsis (Figure 6A). GSEA further confirmed a marked enrichment of the JAK–STAT signalling pathway in the same transcriptomic profiles (Figure 6B), and previous studies have reported that this pathway is involved in numerous inflammation‐related processes.^41^, ^42^, ^43^ We subsequently analysed cardiac tissues from both WT and KO SIMD mice, as well as from Col1a2‐iCre and flox/flox SIMD mice, using WB. The results revealed that cardiac tissues from SIMD mice displayed markedly elevated Jak1 and Stat3 phosphorylation relative to control animals. Furthermore, among septic mice, Serpina3n KO and Col1a2‐iCre animals exhibited stronger Jak1 and Stat3 phosphorylation than their WT and flox/flox counterparts, respectively (Figure 6C,D). Additionally, we analysed cardiac tissues from CLP‐induced WT mice, Serpina3n KO mice, flox/flox mice and Serpina3n Col1a2‐iCre mice. These CLP models recapitulated the phenotypic alterations observed in the LPS‐induced sepsis model (Figure S11A,B).

**FIGURE 6:**
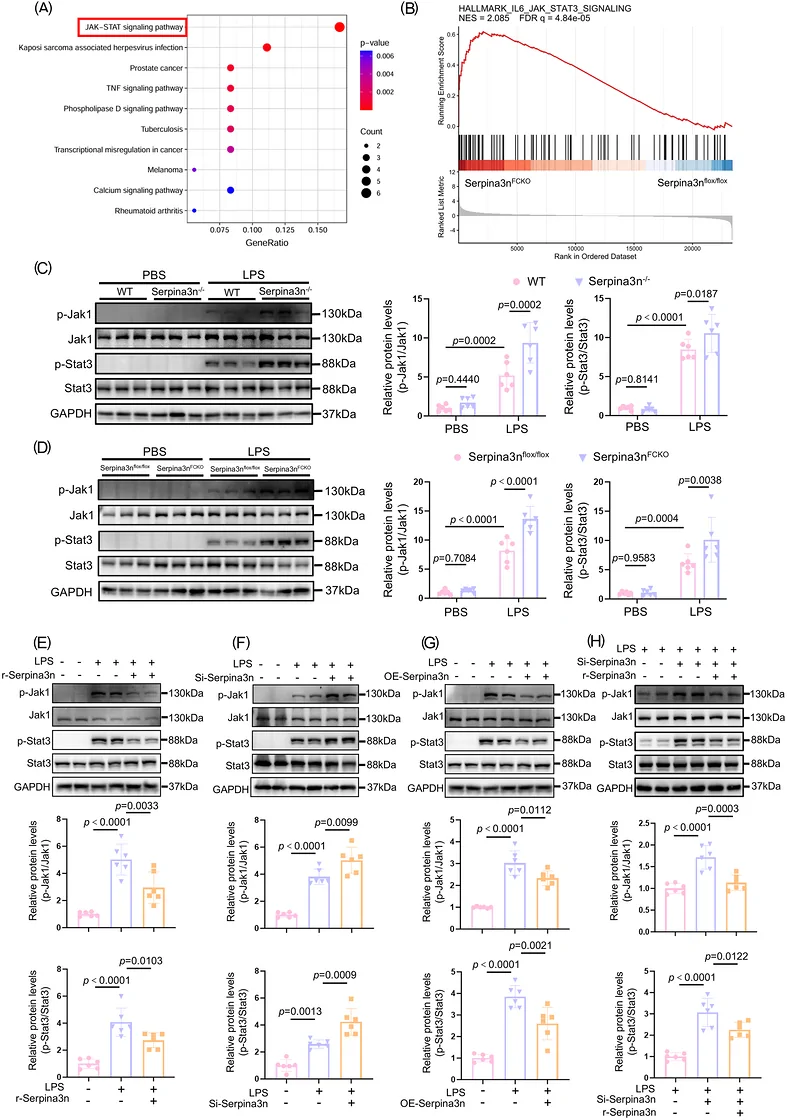
Serpina3n is involved in CF–CM crosstalk and protects CMs by disrupting the JAK–STAT pathway. (A) The results of the KEGG enrichment analysis showed that the JAK–STAT pathway was significantly activated in Col1a2‐iCre mice with sepsis. (B) GSEA of the transcriptomic profiles in Col1a2‐iCre mice with sepsis. (C) Abundance of proteins related to the JAK–STAT pathway in the cardiac tissues of WT and Serpina3n^−/−^ mice at 12 h after LPS administration. (D) Abundance of proteins related to the JAK–STAT pathway in the cardiac tissues of flox/flox and Col1a2‐iCre mice at 12 h after LPS administration. (E) Treatment with r‐Serpina3n attenuated the LPS‐induced phosphorylation of Jak1 and Stat3 in NMCMs. (F) Knockdown of Serpina3n in NMCFs resulted in increased phosphorylation of Jak1 and Stat3 in NMCMs. (G) Overexpression of Serpina3n in NMCFs reduced the LPS‐induced phosphorylation of Jak1 and Stat3 in NMCMs. (H) Abundances of the Jak1, p‐Jak1, Stat3 and p‐Stat3 proteins in NMCMs after treatment with conditioned medium from Serpina3n‐knockdown NMCFs, with or without r‐Serpina3n rescue. Densitometric quantification of the Western blots in (C–H) was performed using ImageJ, with GAPDH serving as a loading control. All the data are shown as the mean ± SD. Data shown in (C and D) represent *n* = 6 mice per group, and data in (E–H) represent *n* = 6 independent biological replicates. Statistical analyses of the data presented in (E–H) were performed using one‐way ANOVA with Tukey's post hoc test. For the analysis of the data in (C) and (D), two‐way ANOVA was applied. *Abbreviations*: FCKO, fibroblast‐specific Serpina3n KO; r‐Serpina3n, recombinant mouse Serpina3n protein; Si‐Serpina3n, Serpina3n siRNA; OE‐Serpina3n, Serpina3n overexpression.

We then validated these findings in vitro. Alterations in JAK–STAT pathway‐related protein expression were evaluated; one group of NMCMs was stimulated with LPS, while another group was treated with r‐Serpina3n at the time of LPS stimulation. The findings showed that r‐Serpina3n treatment reversed the considerable increases in Jak1 and Stat3 phosphorylation in NMCMs caused by LPS stimulation (Figure 6E). We further examined the nuclear translocation of Stat3 by WB. The results revealed that LPS stimulation markedly promoted the nuclear translocation of phosphorylated Stat3, whereas treatment with r‐Serpina3n efficiently alleviated this LPS‐induced effect (Figure S11C). In addition, we cultured NMCMs in conditioned medium from fibroblasts treated with Si‐Serpina3n or OE‐Serpina3n. The knockdown of Serpina3n in NMCFs significantly increased the LPS‐induced phosphorylation of Jak1 and Stat3 in NMCMs (Figure 6F). In contrast, Serpina3n overexpression suppressed Jak1 and Stat3 phosphorylation in NMCMs (Figure 6G). We further performed rescue experiments by adding r‐Serpina3n to the culture medium of Serpina3n knockdown NMCFs. The collected conditioned medium was subsequently used to treat the NMCMs. As expected, Serpina3n silencing caused a substantial up‑regulation of p‑Jak1/p‑Stat3 in NMCMs, whereas r‑Serpina3n treatment abrogated this change (Figure 6H). These data confirm that CMs themselves are capable of responding to LPS stimulation and activating the JAK–STAT signalling pathway.

We further confirmed that the JAK–STAT signalling cascade mediates the cardioprotective effects of Serpina3n by applying a specific Stat3 inhibitor (#HY‐15146; MedChemExpress, Monmouth Junction, New Jersey, USA) to determine whether it could mimic the protective effect of r‐Serpina3n on LPS‐challenged NMCMs. We first verified that the concentration of the Stat3 inhibitor used in our study (10 µM) did not affect NMCM viability (Figure S12A). Pharmacological inhibition of Stat3 expression significantly reduced the mRNA levels of pro‐inflammatory cytokines and decreased Stat3 phosphorylation upon LPS stimulation, recapitulating the protective effects of r‐Serpina3n (Figure S12B–E).

In contrast, a Stat3 activator (#HY‐P1061; MedChemExpress) was used to verify whether excessive Stat3 activation could abrogate Serpina3n‐mediated protection. Similarly, we first confirmed that the concentration of Stat3 activator used (1 µM) had no impact on NMCM viability (Figure S12F). Notably, Stat3 activator treatment further increased the expression of pro‐inflammatory cytokines and increased Stat3 phosphorylation in LPS‐treated NMCMs, thereby reversing the anti‐inflammatory and cardioprotective effects of r‐Serpina3n (Figure S12G–J).

These findings indicated that the JAK–STAT pathway plays a crucial role in LPS‐induced cardiac dysfunction and myocardial inflammatory cell infiltration, and that the secretion of Serpina3n from CFs may mediate CF–CM crosstalk and exert cardioprotective effects by inhibiting this pathway.

## 4 DISCUSSION

In this study, we revealed that the up‐regulated expression of Serpina3n under conditions of sepsis originates primarily from CFs and mediates CF–CM crosstalk, leading to anti‐inflammatory and cardioprotective actions against SIMD. Mechanistically, our results indicate that Stat3 directly binds to the Serpina3n promoter and increases its transcription in fibroblasts, thereby increasing the secretion of Serpina3n from CFs, which acts on CMs and inhibits the JAK–STAT pathway. These findings identify a previously unrecognised paracrine axis in which fibroblast‐derived Serpina3n limits excessive cardiac inflammation during sepsis (Figure 7).

**FIGURE 7:**
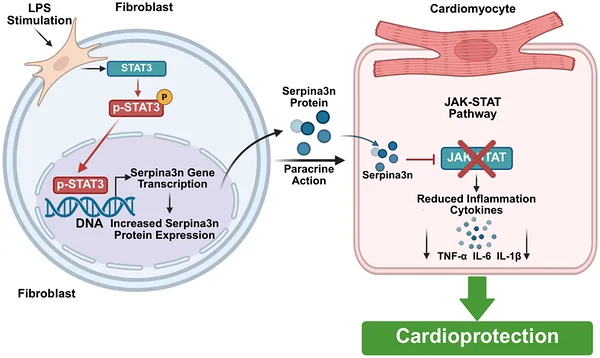
Schematic illustration of this study. LPS‐induced STAT3 phosphorylation in cardiac fibroblasts promotes STAT3 nuclear translocation, increases Serpina3n transcriptional activity and induces Serpina3n up‐regulation and secretion. Secreted Serpina3n then acts on cardiomyocytes via paracrine signalling, inhibits the JAK–STAT pathway, lowers mRNA expression of pro‐inflammatory cytokines including TNF‑α, IL‑6 and IL‑1β and ultimately exerts cardioprotective effects.

SerpinA3 (human) and Serpina3n (mouse) have each been implicated in the development of diverse cardiovascular diseases. Sun et al. reported that the genetic ablation of Serpina3n resulted in significant increases in myocardial fibrosis, cardiac hypertrophy and inflammatory responses following myocardial infarction.^27^ By comparison, Zhang and colleagues demonstrated that SerpinA3 aggravates myocardial infarction within rat and cell‐based experimental models,^44^ indicating potential context‐specific or species‐dependent effects. Notably, while these studies focused on ischemic injury, our work extends this literature by establishing a protective role for fibroblast‐derived Serpina3n in SIMD—a distinct pathophysiological setting characterised by systemic inflammation rather than focal ischemia. Moreover, we provide the first evidence that Serpina3n acts as a paracrine mediator between fibroblasts and CMs rather than functioning solely as an intracellular or systemic factor. Although it remains unclear in human studies whether SerpinA3 expression is a pathogenic factor or a compensatory response, our loss‐ and gain‐of‐function animal and cell models provide evidence that Serpina3n up‐regulation is protective in SIMD, consistent with its association with anti‐inflammatory signatures in a broad spectrum of pathological conditions.^45^, ^46^, ^47^

The JAK–STAT pathway is a well‐established inflammatory cascade that, when excessively or persistently activated, drives maladaptive inflammation, fibrosis, heart failure, CM apoptosis and matrix remodelling.^48^, ^49^ Tight regulation of this pathway is achieved through multiple mechanisms, including SOCS3‐mediated negative feedback and microRNA‐mediated suppression.^50^, ^51^ In our study, Serpina3n attenuated LPS‐induced JAK–STAT activation in CMs and reduced the mRNA levels of downstream inflammatory cytokines (TNF‐α, IL‐6 and IL‐1β). These findings identify Serpina3n as a novel extracellular regulator of this pathway, expanding the known repertoire of JAK–STAT modulators beyond the classical intracellular negative‐feedback loops. However, two important unresolved questions remain. First, given that Serpina3n is a secreted protein, how does it enter CMs to modulate intracellular JAK–STAT signalling? We propose that Serpina3n may be internalised by CMs via receptor mediated endocytosis, a mechanism well documented for other Serpin family members.^52^ For instance, LDL receptor‐related protein 1 (LRP1) serves as a major endocytic receptor for multiple serpin–enzyme complexes, including antithrombin III–thrombin and α1‐antitrypsin–trypsin complexes.^53^ The SerpinB family member vaspin is internalised through LRP1‐dependent, clathrin‐mediated endocytosis^54^ and SerpinE2 has been shown to be internalised by CFs and CMs through a direct interaction with LRP1.^55^ More relevantly, Serpina3k (a close homolog of Serpina3n) binds to LRP6 with high affinity and is internalised via LRP6‐mediated endocytosis, where it functions as an endogenous Wnt antagonist.^56^ Based on these precedents, we speculate that Serpina3n may enter CMs through a similar LRP‑mediated endocytic pathway, although the specific receptor involved remains to be identified. Second, even if Serpina3n is internalised, it remains unclear how it suppresses intracellular JAK–STAT phosphorylation after entering CMs. Based on previous studies,^57^, ^58^, ^59^ we hypothesise that once internalised, Serpina3n may directly interact with Jak1 or Stat3 or indirectly interfere with the JAK–STAT signalling cascade through other intermediates. We acknowledge these as key limitations and future studies are warranted to identify the putative receptor and the precise molecular mechanism underlying Serpina3n mediated JAK–STAT suppression in CMs.

The therapeutic implications of our findings should be interpreted with appropriate caution. Although our data reveal that Serpina3n exerts cardioprotective effects on a mouse model of SIMD and that recombinant Serpina3n suppresses inflammatory responses in cultured CMs, we have not yet established that exogenous Serpina3n administration is an effective therapy for SIMD in vivo, and we recognise that there remains insufficient evidence to confirm the protective function of Serpina3n. The translational potential of Serpina3n‑based strategies therefore remains at an early experimental stage, and further rigorous in vivo pharmacological studies are required. Additionally, the relationship between mouse Serpina3n and human SerpinA3 requires careful interpretation. Although the mouse Serpina3 gene family includes multiple paralogs (e.g., Serpina3n, Serpina3c, Serpina3k) that do not share simple one‐to‐one orthology with human SerpinA3, cumulative expression, structural and biochemical evidence identifies Serpina3n as the functional ortholog of human SerpinA3, whereas the other paralogs have diverged in function.^60^, ^61^, ^62^ Therefore, our findings using Serpina3n may provide relevant insights into the potential role of human SerpinA3 in SIMD, although the existence of multiple Serpina3 paralogs in mice cautions against direct translational extrapolation.

The novelty of the present work resides in three major dimensions. First, this study provides the first evidence establishing the protective role of fibroblast‐derived Serpina3n in SIMD, thereby filling a critical knowledge gap regarding the function of this protein in the context of cardiovascular inflammation. Second, we characterise Serpina3n as an upstream suppressor of JAK–STAT signalling in CMs. Third, we identify a novel paracrine fibroblast–CM signalling axis regulated by Serpina3n.

There are several limitations to this study. First, the interaction between Serpina3n and other inflammatory pathways, such as the MAPK and TLR4/NF‑κB cascades, was not investigated; thus, the anti‑inflammatory mechanism of Serpina3n requires further validation through more in‑depth molecular interaction experiments. Second, our findings are derived primarily from cell and mouse models, and whether SerpinA3 expression correlates with clinical outcomes in patient samples has not been verified. Third, as Serpina3n is a secreted protein, whether it exerts autocrine effects on cardiac fibroblasts—such as regulating Stat3 signalling and Stat3‐mediated pro‐inflammatory cytokine expression in cardiac fibroblasts—was not experimentally addressed in the present study and warrants further investigation. Fourth, the effects of Serpina3n on other pathological processes involved in SIMD, including CM apoptosis and oxidative stress, were not explored in this study, and the specific cell surface receptor(s) for Serpina3n as well as its precise intracellular mechanism remain to be identified. Fifth, although our in vitro data demonstrate that Serpina3n suppresses JAK–STAT signalling in CMs, it remains unclear whether this effect is cell‑type‑specific or extends to other cardiac cell populations, and the differential expression of Serpina3n between cardiac fibroblasts and CMs together with its cell‐type‐specific regulatory mechanisms warrants further investigation. Sixth, all animal experiments were performed on male mice. Given the well‑documented sex differences in immune responses, inflammatory signalling and cardiac pathology,^63^, ^64^, ^65^ whether the observed protective role of Serpina3n in SIMD extends to female subjects remains to be established. In addition, the lack of mouse lines that support exclusive gene knockout in cardiac fibroblasts represents a technical limitation; consequently, the Col1a2‑iCre driver employed in this study exhibits broad targeting of fibroblasts across multiple tissues. Future studies are needed to address these gaps.

In summary, our findings establish a protective paracrine role for fibroblast‐derived Serpina3n in limiting JAK–STAT‐mediated inflammation during sepsis, providing a mechanistic framework and experimental foundation for future studies exploring Serpina3n‐based interventions. However, substantial translational hurdles remain, including the lack of in vivo efficacy data for exogenous Serpina3n delivery, species differences between mouse Serpina3n and human SerpinA3, and the need for clinical validation. Addressing these gaps will be essential before Serpina3n can be considered a viable therapeutic target for SIMD.

## AUTHOR CONTRIBUTIONS

G. F. Z., Z. Y. H. and H. X. conceived this study and helped with the experimental design. X. N. W. and Y. F. Y. contributed equally to this work, including data collection and drafting of the original manuscript. A. Q. K. and X. Y. L. assisted in the data analysis and provided statistical support, ensuring the reliability and accuracy of interpretation of the results. The project was overseen by G. F. Z., Z. Y. H. and H. X., who also made revisions to the manuscript. Before submission, the final draft of the manuscript was reviewed and approved by each author.

## CONSENT

The authors have nothing to report.

## CONFLICT OF INTEREST STATEMENT

The authors declare no conflicts of interest.

## ETHICS STATEMENT

Every animal experiment was carried out in accordance with the rules established by Nanjing Medical University's Animal Care and Use Committee (ethical approval number DWSY25133771).

## Supporting information



Supporting Information

## Data Availability

Data backing the conclusions drawn in this investigation may be obtained from the corresponding author following reasonable requests.
