# Supplementary material for: Serpina3n deficiency exacerbates sepsis‐induced myocardial dysfunction by disrupting fibroblast–cardiomyocyte crosstalk
Source: Clin Transl Med. 2026 Nov 14;16(11):e70898. doi: 10.1002/ctm2.70898 (PMC13770213; doi:10.1002/ctm2.70898)
Supplement: Supplementary file 1 [file CTM2-16-e70898-s001.docx]

Supporting Information

**Serpina3n deficiency exacerbates** **sepsis-induced myocardial dysfunction by disrupting fibroblast–cardiomyocyte crosstalk**

Xunuo Wang^†^, Yufeng Yan^†^, Aiqing Kong, Xiya Li, Hao Xie*, Zuoying Hu*, Guangfeng Zuo*

Department of Cardiology, Nanjing First Hospital, Nanjing Medical University, Nanjing, China

†Xunuo Wang and Yufeng Yan contributed equally to this work.

*Correspondence:

Guangfeng Zuo, Department of Cardiology, Nanjing First Hospital, Nanjing Medical University, 68 Changle Road, Qinhuai, Nanjing 210006, China. E-mail address: anlizgf@njmu.edu.cn

Zuoying Hu, Department of Cardiology, Nanjing First Hospital, Nanjing Medical University, 68 Changle Road, Qinhuai, Nanjing 210006, China. E-mail address: [zuoying_hu@126.com](mailto:zuoying_hu@126.com)

Hao Xie, Department of Cardiology, Nanjing First Hospital, Nanjing Medical University, 68 Changle Road, Qinhuai, Nanjing 210006, China. E-mail address: xie_hao@njmu.edu.cn

**Methods**

**GEO database**

**Bulk RNA‑seq analysis (Bulk RNA‑seq)**

Bulk RNA-seq datasets used in this study were downloaded from the Gene Expression Omnibus (GEO) database (https://www.ncbi.nlm.nih.gov/geo/). All three datasets comprise mouse cardiac tissue samples. GSE267388 contains a total of 10 samples (5 PBS controls and 5 LPS-treated samples); GSE215955 includes 12 samples (4 PBS controls, 4 LPS-treated samples, and 4 LPS + XJDHT treatment samples); GSE229925 consists of 15 samples (4 sham-operated controls and 11 CLP-induced SIMD samples). Rigorous data preprocessing and quality control were performed prior to differential expression analysis. Briefly, raw intensities were subjected to background correction and normalization using the Robust Multi-array Average (RMA) method. Probe IDs were mapped to gene symbols based on the platform annotation file. Probes mapping to multiple genes were discarded, whereas for genes targeted by multiple probes, the probe with the highest average expression value was retained. Low-expression genes (e.g., raw count < 10 or TPM < 1 in more than 80% of samples) were filtered out. Potential outlier samples were detected and excluded by principal component analysis (PCA) to ensure the robustness of downstream analyses.

Accession link for GSE215955: https://www.ncbi.nlm.nih.gov/geo/query/acc.cgi?acc=GSE215955

Accession link for GSE229925: https://www.ncbi.nlm.nih.gov/geo/query/acc.cgi?acc=GSE229925

Accession link for GSE267388: https://www.ncbi.nlm.nih.gov/geo/query/acc.cgi?acc=GSE267388

**Single-cell RNA-seq analysis (scRNA-seq)**

The single-cell transcriptomic data utilized in this study were retrieved from two public datasets: Dataset A (GSE207363) and Dataset B (GSE285720). Each dataset was processed independently, including quality control, clustering and downstream analyses. The robustness of our results was validated by comparing cell subpopulation distributions and functional characteristics within each dataset.

We implemented stringent filtering criteria: cells were excluded if they possessed (1) < 500 or > 60,000 UMIs, (2) < 200 or > 8,000 detected genes, or (3) > 5% mitochondrial gene content. DoubletFinder was used to remove doublets. For data normalization, we employed the pp.normalize_total function (scaling factor = 10,000), followed by log-transformation via pp.log1p. After highly variable genes were identified using the method described by Macosko et al,^1^ we performed principal component analysis (PCA) for dimensionality reduction. To mitigate technical variation and batch effects across samples, the Harmony algorithm was applied in the PCA space to integrate the datasets. The clustering resolution was set to 0.3 for GSE207363 and 0.2 for GSE285720. A nearest-neighbor graph was subsequently constructed to enable graph-based clustering, and the results were visualized in two dimensions using UMAP.

The FindAllMarkers function (Wilcoxon rank-sum test) in the Seurat R package was used to identify marker genes and differentially expressed genes (DEGs) for each cluster. Genes were considered significant if the adjusted P value < 0.05 and |log₂(fold change)| > 0.25, with an additional requirement that the gene was detected in at least 10% of cells in either group (min.pct = 0.1). The cell counts of GSE207363 and GSE285720 are listed in Table S4 and Table S5, respectively.

Cell–cell communication analysis was performed using the R package CellChat.^2^ The normalized and cell-type annotated Seurat object was used as input, and the standard mouse ligand–receptor interaction database (CellChatDB.mouse) was adopted as the reference. Communication probabilities were calculated and filtered to evaluate signaling pathway activity. Network diagrams, bubble plots and chord diagrams were finally generated to visualize global communication patterns and ligand–receptor pairs of specific cell populations.

The pySCENIC algorithm was applied to infer transcription factor regulatory networks at the single-cell level.^3^ First, the GRNBoost2 algorithm was used to calculate gene co-expression weights based on the single-cell expression matrix and construct co-expression modules consisting of transcription factors and their putative target genes. The cisTarget algorithm combined with the mouse-specific motif database (mm10) was then utilized for cis-regulatory element enrichment analysis to filter false-positive targets lacking evidence of transcription factor binding, yielding high-confidence regulatory units (Regulons). Finally, the AUCell algorithm computed the enrichment score of Regulon target genes within each single cell to generate a matrix of single-cell Regulon activity scores, which was used to assess heterogeneity in transcription factor regulation across distinct cell states.

Accession link for GSE285720: https://www.ncbi.nlm.nih.gov/geo/query/acc.cgi?acc=GSE285720

Accession link for GSE207363: https://www.ncbi.nlm.nih.gov/geo/query/acc.cgi?acc=GSE207363

**FIGURE S1**

**FIGURE S1** Cell purity was validated by immunofluorescence staining. Scale bars: 100 μm.

**FIGURE S2**

**FIGURE S2** Single‑cell data analysis of the GSE207363 database. (A) Annotation analysis on the markers of each cell. (B) Violin plot showing that Serpina3n is predominantly expressed in fibroblasts. (C) The expression of Serpina3n in cardiac fibroblasts with or without SIMD.

**FIGURE S3**

**FIGURE S3** Single‑cell data analysis of the GSE285720 database. (A) The expression of Serpina3n in cardiac fibroblasts with or without SIMD. (B) Violin plot showing that Serpina3n is predominantly expressed in fibroblasts. (C) UMAP showing the presence of eleven distinct heart cell types. (D) The percentage of each cell type in mice with or without SIMD. (E) Annotation analysis on the markers of each cell. (F) Co-localization of Serpina3n gene with fibroblast marker genes.

**FIGURE S4**

**FIGURE S4** Serpina3n is predominantly derived from cardiac fibroblasts and acts on cardiomyocytes in a paracrine manner. (A) Immunofluorescence staining of Serpina3n with cell-specific markers for cardiomyocytes, endothelial cells, macrophages and neutrophils. Scale bars: 100 μm. (B) ELISA analysis of Serpina3n levels in supernatants from NMCFs, NMCMs, and NMECs. (C) Schematic diagram illustrating the NMCF–NMCM co‑culture system, together with Serpina3n protein abundance and mRNA levels in NMCMs before and after co‑culture with NMCFs. Densitometric quantification of the Western blots was performed using ImageJ, with GAPDH serving as a loading control. All the data are shown as the mean ± SD. Data shown in (B, C) represent *n* = 6 independent biological replicates. Statistical analyses of the data presented in (C) were performed using one-way ANOVA with Tukey’s post hoc test. For the analysis of the data in (B), two‑way ANOVA was applied. *Abbreviations*: NMCFs, primary neonatal mouse cardiac fibroblasts; NMCMs, primary neonatal mouse cardiomyocytes. NMECs, primary neonatal mouse cardiac endothelial cells.

**FIGURE S5**

**FIGURE S5** The loss of Serpina3n deteriorates cardiac function and inflammation in the hearts of mice with CLP-induced sepsis. (A) Survival curves of WT and Serpina3n^-/-^ mice with or without CLP. (B) Representative echocardiograms of WT and Serpina3n^-/-^ mice at 12 hours after CLP administration. (C–F) The ejection fraction (EF), fractional shortening (FS), E/A ratio, and isovolumic relaxation time (IVRT) were quantified in the columns. (G–I) Relative mRNA levels of inflammatory factors in the cardiac tissues of WT and Serpina3n^-/-^ mice after CLP administration. (J) Representative images of CD11b and Ly6G staining in cardiac tissue sections from the experimental mice. Scale bars: 100 μm. All the data are shown as the mean ± SD. Data shown in (A) represent *n* = 16 mice per group, and data in (C–J) represent *n* = 6 mice per group. Statistical analyses of the data presented in (G–I) were performed using one-way ANOVA with Tukey’s post hoc test. For the analysis of the data in (C–F) and (J), two‑way ANOVA was applied. *Abbreviation*: CLP, cecal ligation and puncture.

**FIGURE S6**

**FIGURE S6** Protein abundance of Serpina3n in cardiac cells from mice subjected to LPS stimulation. (A) Protein abundance of Serpina3n in cardiac fibroblasts from flox/flox and Serpina3n Col1a2-iCre mice after LPS stimulation. (B) Serpina3n protein expression in AMCFs, AMCMs, and ECs from Col1a2-iCre mouse hearts. Densitometric quantification of the Western blots was performed using ImageJ, with GAPDH serving as a loading control. All the data are shown as the mean ± SD. Data shown in (A, B) represent *n* = 6 mice per group. Statistical analyses of the data were performed using one-way ANOVA with Tukey’s post hoc test. *Abbreviations*: FCKO, fibroblast-specific Serpina3n KO; AMCFs, adult mouse cardiac fibroblasts; AMCMs, adult mouse cardiomyocytes; ECs, endothelial cells.

**FIGURE S7**

**FIGURE S7** Fibroblast-specific Serpina3n deficiency induces cardiac dysfunction and inflammation in the hearts of mice with CLP-induced sepsis. (A) Survival curves of flox/flox and Serpina3n Col1a2-iCre mice with or without CLP. (B) Representative echocardiograms of flox/flox and Serpina3n Col1a2-iCre mice at 12 hours after CLP. (C–F) The ejection fraction (EF), fractional shortening (FS), E/A ratio, and isovolumic relaxation time (IVRT) were quantified in the columns. (G–I) Relative mRNA levels of inflammatory factors in the cardiac tissues of flox/flox and Serpina3n Col1a2-iCre mice after CLP administration. (J) Representative images of CD11b and Ly6G staining in cardiac tissue sections from the experimental mice. Scale bars: 100 μm. All the data are shown as the mean ± SD. Data shown in (A) represent *n* = 16 mice per group, and data in (C–J) represent *n* = 6 mice per group. Statistical analyses of the data presented in (G), (H), and (I) were performed using one-way ANOVA with Tukey’s post hoc test. For the analysis of the data in (C–F) and (J), two‑way ANOVA was applied. *Abbreviations*: CLP, cecal ligation and puncture; FCKO, fibroblast-specific Serpina3n KO.

**FIGURE S8**

**FIGURE S8** Serpina3n is internalized by NMCMs and protects NMCMs against LPS-induced cellular injury. (A) Western blot detection of intracellular Flag‑Serpina3n and His‑Serpina3n after uptake by NMCMs. Acid wash eliminated membrane-attached protein, and 4 °C incubation was used to inhibit active endocytosis. (B) Cell viability and LDH release of NMCMs upon LPS stimulation with or without r‑Serpina3n treatment. Densitometric quantification of the Western blots was performed using ImageJ, with GAPDH serving as a loading control. All the data are shown as the mean ± SD. Data shown in (A, B) represent *n* = 6 independent biological replicates. Statistical analyses of the data presented in (B) were performed using one-way ANOVA with Tukey’s post hoc test. *Abbreviations*: Flag–Serpina3n, Flag-tagged recombinant Serpina3n protein; His–Serpina3n, His-tagged recombinant Serpina3n protein; r-Serpina3n, recombinant mouse Serpina3n protein.

**FIGURE S9**

**FIGURE S9** Transcriptional regulatory analysis of the single‑cell database GSE285720. (A) Gene expression levels of transcription factors in each cell type shown by the dot plot. (B) Expression levels of various transcription factors in different cell types shown by the heatmap.

**FIGURE S10**

**FIGURE S10** Stat3 promotes the transcription of Serpina3n in NMCFs. (A) mRNA expression of Stat3 in NMCFs after Stat3 plasmid transfection. (B) mRNA expression of Serpina3n in NMCFs after Stat3 plasmid transfection. (C) Protein abundance of p-Stat3, Stat3, Serpina3n in NMCFs after Stat3 plasmid transfection. Densitometric quantification of the Western blots was performed using ImageJ, with GAPDH serving as a loading control. All the data are shown as the mean ± SD. Data shown in (A–C) represent *n* = 6 independent biological replicates. Statistical analyses of the data were performed using one-way ANOVA with Tukey’s post hoc test. *Abbreviation*: OE-Stat3: Stat3 overexpression.

**FIGURE S11**

**FIGURE S11** Serpina3n deficiency activates JAK–STAT signaling and promotes Stat3 nuclear translocation. (A) Abundance of proteins related to the JAK–STAT pathway in the cardiac tissues of WT and Serpina3n^-/-^ mice at 12 hours after CLP administration. (B) Abundance of proteins related to the JAK–STAT pathway in the cardiac tissues of flox/flox and Col1a2-iCre mice at 12 hours after CLP administration. (C) Nuclear and cytoplasmic p‑Stat3 and Stat3 protein levels in NMCMs. Lamin B1 and GAPDH served as fractionation controls. Relative nuclear p‑Stat3 levels were quantified by densitometry and normalized to Lamin B1. Densitometric quantification of the Western blots was performed using ImageJ, with GAPDH and Lamin B1 serving as loading controls. All the data are shown as the mean ± SD. Data shown in (A, B) represent n = 6 mice per group, and data in (C) represent *n* = 6 independent biological replicates. Statistical analyses of the data in (C) were performed using one-way ANOVA with Tukey’s post hoc test. For the analysis of the data in (A), and (B), two-way ANOVA was applied. *Abbreviations*: CLP, cecal ligation and puncture; FCKO, fibroblast-specific Serpina3n KO; r-Serpina3n, recombinant mouse Serpina3n protein.

**FIGURE S12**

**FIGURE S12** Stat3 inhibition mimics while Stat3 activation abrogates the protective effects of r‑Serpina3n in LPS‑stimulated NMCMs**.** (A) Cell viability of NMCMs following treatment with 10 μM S3I-201 for 12 hours. (B–D) Relative mRNA levels of TNF‑α, IL‑6, and IL‑1β in LPS‑stimulated NMCMs treated with or without a Stat3 inhibitor. (E) Western blot analysis and quantification of p‑Stat3/Stat3 ratio under the same conditions. (F) Cell viability of NMCMs following treatment with 1 μM Colivelin for 12 hours. (G–I) Relative mRNA levels of TNF‑α, IL‑6, and IL‑1β in LPS‑stimulated NMCMs treated with r‑Serpina3n and/or a Stat3 activator. (J) Western blot analysis and quantification of p‑Stat3/Stat3 ratio under the same conditions. Densitometric quantification of the Western blots was performed using ImageJ, with GAPDH serving as a loading control. All the data are shown as the mean ± SD. Data shown in (A–J) represent *n* = 6 independent biological replicates. Statistical analyses of the data in (A–F) were performed using one-way ANOVA with Tukey’s post hoc test. For the analysis of the data in (G–J), two-way ANOVA was applied. *Abbreviation*: r-Serpina3n, recombinant mouse Serpina3n protein.

**Table S1. Primer sequences for reverse-transcription quantitative PCR (qPCR)**

| **Gene Name** | **Primer Sequence** |
| --- | --- |
| Serpina3n | Forward: 5’-ATTTGTCCCAATGTCTGCGAA-3’  Reverse:5’-TGGCTATCTTGGCTATAAAGGGG-3’ |
| 18s | Forward: 5’-GGCCGTTCTTAGTTGGTGGAGCG-3’  Reverse:5’-CTGAACGCCACTTGTCCCTC-3’ |
| TNF-α | Forward: 5’-CAGGCGGTGCCTATGTCTC-3’  Reverse:5’-CGATCACCCCGAAGTTCAGTAG-3’ |
| IL-1β | Forward: 5’-GCAACTGTTCCTGAACTCAACT-3’  Reverse:5’-ATCTTTTGGGGTCCGTCAACT-3’ |
| IL-6 | Forward: 5’-AGTCCGGAGAGGAGACTTCA-3’  Reverse: 5’-TTCCACGATTTCCCAGAG-3’ |
| Stat3 | Forward: 5’-CACCTTGGATTGAGAGTCAAGAC-3’  Reverse: 5’-AGGAATCGGCTATATTGCTGGT-3’ |

**Table S2. Sequence information of siRNAs used in RNA knockdown assay.**

| **Gene Name** | **Primer Sequence** |
| --- | --- |
| Scramble | Sense: 5’-UUCUCCGAACGUGUCACGUTT-3’  Antisense:5’-ACGUGACACGUUCGGAGAATT-3’ |
| Serpina3n | Sense: 5’-GGAGUCAAAUUUGUCCCAAUGTT-3’  Antisense: 5’-CAUUGGGACAAAUUUGACUCCTT-3’ |

**Table S3. Primer sequences for qPCR of chromatin immunoprecipitation (ChIP) assays**

| **Promoter** | **Primer Sequence** |
| --- | --- |
| Serpina3n-ChIP-qPCR1 | Forward: 5’-GTGGTTAAACACACTCACTGCTC-3’ |
|  | Reverse:5’-GAGTACACTGCAGCTGTTTTCAG-3’ |
| Serpina3n-ChIP-qPCR2 | Forward: 5’-GCCATGATATTCCTACCCGACAG-3’ |
|  | Reverse:5’-GAGCAACTCTGTCTGTCCTTCCAG-3’ |
| Serpina3n-ChIP-qPCR3 | Forward: 5’-CTGGAAGGACAGACAGAGTTGC-3’ |
|  | Reverse:5’-CTGCTGTACTGGACAAGTCTTCTG-3’ |
| Serpina3n-ChIP-qPCR4 | Forward: 5’-GTGCACTTTCCTTTTGCTTCTGAC-3’ |
|  | Reverse:5’-GCTGTGCTCTGTGCTCAGAC-3’ |

**Table S4. Cell counts for dataset GSE207363**

| Cell Type | Ctrl | SICM |
| --- | --- | --- |
| Endothelial Cells | 3721 | 2264 |
| Macrophages | 4213 | 1762 |
| Fibroblasts | 2202 | 2093 |
| T cells | 940 | 368 |
| Smooth Muscle | 468 | 179 |
| Pericytes | 674 | 289 |
| Cardiomyocytes | 222 | 230 |
| Neutrophils | 190 | 235 |
| Endocardial Endothelial Cells | 227 | 102 |
| B cells | 245 | 34 |
| Lymphatic Endothelial Cells | 168 | 84 |
| Schwann Cells | 182 | 63 |
| Proliferating Cells | 134 | 21 |

SICM: septic cardiomyopathy

**Table S5. Cell counts for dataset GSE285720**

| Cell Type | Ctrl | SICM |
| --- | --- | --- |
| Activated Fibroblasts | 3 | 6521 |
| Endothelial Cells | 1481 | 1210 |
| Resident Fibroblasts | 1872 | 1580 |
| Endocardial Cells | 1081 | 507 |
| Macrophages | 529 | 569 |
| Pericytes | 394 | 354 |
| Lymphatic Endothelial | 124 | 63 |
| Smooth Muscle Cells | 45 | 31 |
| Epicardial Cells | 57 | 13 |
| Schwann Cells | 18 | 27 |
| Cardiomyocytes | 1364 | 996 |

SICM: septic cardiomyopathy

**References:**

1.	Macosko EZ, Basu A, Satija R, et al. Highly parallel genome-wide expression profiling of individual cells using nanoliter droplets. *Cell*. May 21 2015;161(5):1202–1214. doi:10.1016/j.cell.2015.05.002

2.	Jin S, Guerrero-Juarez CF, Zhang L, et al. Inference and analysis of cell-cell communication using CellChat. *Nat Commun*. Feb 17 2021;12(1):1088. doi:10.1038/s41467-021-21246-9

3.	Aibar S, González-Blas CB, Moerman T, et al. Scenic: Single-cell regulatory network inference and clustering. *Nat Methods*. Nov 2017;14(11):1083–1086. doi:10.1038/nmeth.4463
